# Exogenous regulation of macronutrients promotes the accumulation of alkaloid yield in *anisodus tanguticus* (Maxim.) pascher

**DOI:** 10.1186/s12870-024-05299-8

**Published:** 2024-06-26

**Authors:** Na Liu, Chen Chen, Bo Wang, Xiaoyun Wang, Dengshan Zhang, Guoying Zhou

**Affiliations:** 1 Northwest Institute of Plateau Biology, CAS Key Laboratory of Tibetan Medicine Research, Xining, 810008 China; 2 State Key Laboratory of Plateau Ecology and Agriculture, Qinghai University, Xining, 810016 China; 3 College of Life Sciences, Huaibei Normal University, Huaibei, 235000 China

**Keywords:** Nitrogen, Phosphorus, Potassium, *Anisodus tanguticus*, Tropane alkaloids, Douglas production function

## Abstract

**Background:**

*Anisodus tanguticus* (Maxim.) Pascher (*A. tanguticus*) is a valuable botanical for extracting tropane alkaloids, which are widely used in the pharmaceutical industry. Implementing appropriate cultivation methods can improve both the quality and yield of *A. tanguticus*. A two-year field experiment was conducted from 2021 to 2023 using a single-factor randomized complete block design replicated three times. The study examined the effects of different nutrient levels (nitrogen: 0, 75, 150, 225, 300, 375 kg/ha; phosphorus: 0, 600, 750, 900, 1050, 1200 kg/ha; potassium: 0, 75, 112.5, 150, 187.5, 225 kg/ha) on the growth, primary alkaloid contents, and alkaloid yield of *A. tanguticus* at different growth stages (S-Greening, S-Growing, S-Wilting; T-Greening, T-Growing, and T-Wilting) in both the roots and aboveground portions.

**Results:**

Our results demonstrate that nutrient levels significantly affect the growth and alkaloid accumulation in *A. tanguticus*. High nitrogen levels (375 kg/ha) notably increased both root and aboveground biomass, while phosphorus had a minimal effect, especially on aboveground biomass. For alkaloid content (scopolamine, anisodamine, anisodine, atropine), a moderate nitrogen level (225 kg/ha) was most effective, followed by low potassium (75 kg/ha), with phosphorus showing a limited impact. Increased phosphorus levels led to a decrease in scopolamine content. During the T-Growing period, moderate nitrogen addition (225 kg/ha) yielded the highest alkaloid levels per unit area (205.79 kg/ha). In the T-Wilting period, low potassium (75 kg/ha) and low phosphorus (750 kg/ha) resulted in alkaloid levels of 146.91 kg/ha and 142.18 kg/ha, respectively. This indicates nitrogen has the most substantial effect on alkaloid accumulation, followed by potassium and phosphorus. The Douglas production function analysis suggests focusing on root biomass and the accumulation of scopolamine and atropine in roots to maximize alkaloid yield in *A. tanguticus* cultivation.

**Conclusions:**

Our findings show that the optimum harvesting period for *A. tanguticus* is the T-Wilting period, and that the optimal nitrogen addition is 225 kg/ha, the optimal potassium addition is 75 kg/ha, and the optimal phosphorus addition is 600 kg/ha or less.

**Supplementary Information:**

The online version contains supplementary material available at 10.1186/s12870-024-05299-8.

## Introduction

*Anisodus tanguticus* (Maxim.) Pascher (*A. tanguticus*) is a perennial herbaceous plant of the family Solanaceae and the genus Scopolamine [1]. This plant is native to the Qinghai-Tibet Plateau [2–5]. The detailed account can be found in China’s Tibetan Medicine Chronicles [6]. The whole plant of *A. tanguticus* contains diverse tropane alkaloids, serving as both an herbal remedy for pain relief and sedation and a vital plant resource for Tibetan herders to mitigate livestock injuries caused by winter’s low temperatures [7]. Being a crucial raw medicinal material, *A. tanguticus* has been extensively studied for its chemical composition, pharmacological activities of monomeric compounds, and its potential as a source for new drugs [8–12]. Since the 1950s, anisodamine has been extracted from the roots of this plant and used as an anticholinergic medication to inhibit M-choline receptors [13–15]. Following that, compounds such as anisodine, scopolamine, and atropine, which are categorized as tropane alkaloids, have been isolated from *A. tanguticus*[16]. Due to distinct substituents at different positions of their molecular structures, these compounds manifest diverse levels of physiological activity [17]. With the rapid expansion of the market for drugs containing alkaloids, many pharmaceutical factories manufacture diverse active pharmaceutical ingredients utilized as mydriatics, antiemetics, antispasmodics, anesthetics, and bronchodilators [18, 19]. Simultaneously, these alkaloids have been listed on the Essential Medicines List of the World Health Organization and received approval from the United States Food and Drug Administration for market sale [12, 20]. Currently, tropane alkaloids are primarily derived from herbal medicines containing such alkaloids [19, 21, 22]. *A. tanguticus*, serving as a crucial plant resource for tropane alkaloid extraction, has evolved from a traditional medicinal plant to an industrial one [10, 18, 23]. As a result, the demand for *A. tanguticus* medicinal materials has steadily risen. Furthermore, given the perennial nature and extended growth cycle of *A. tanguticus*, excessive harvesting has led to the gradual depletion of wild resources, presenting challenges in sustaining the supply for rapidly growing industrial production [16, 24, 25]. Therefore, cultivating *A. tanguticus* stands out as the primary approach to addressing the supply–demand imbalance and concurrently serves as the most effective measure to safeguard the ecological environment.

Accurate fertilizer management is crucial in the pursuit of sustainable development of medicinal plants since it directly affects the quantity and quality of medicinal herbs. Nitrogen (N), phosphorus (P), and potassium (K) are three crucial nutrients that affect the growth, development, and synthesis of bioactive compounds in plants [26]. Optimizing the supply of nitrogen, phosphate, and potassium improves the caliber and productivity of therapeutic herbs. Nitrogen is a crucial component of organic compounds such as proteins, amino acids, and nucleic acids [27]. It plays a vital function in regulating the secondary metabolism of medicinal plants [28]. For instance, when nitrogen is plentiful, plants from the Solanaceae family manifest a notable increase in alkaloids [29, 30]. Similar patterns emerge in other alkaloid compounds like morphine in *Papaver*, scopolamine in *Mandragora*, and quinine in *Cinchona* trees, indicating augmented synthesis under optimal nitrogen supply [31–33]. These alkaloids play essential roles in the medicinal and defensive functions of plants[34, 35]. Also, the right amount of nitrogen has a direct effect on some secondary substances, like celastrol, silymarin, and saponins [36–38]. Hence, the direct regulatory role of nitrogen in the secondary metabolism of plants holds significant importance for the biosynthesis and effectiveness of medicinal plants.

Additionally, phosphorus plays a vital role in numerous cellular processes essential for cell metabolism, division, and plant growth and development[39]. It is a key component of ATP, DNA, RNA, phospholipids, coenzymes, and membrane lipids, functioning as a primary metabolic regulator [40, 41]. An abundant provision of phosphorus in medicinal plants promotes root growth and enhances the production of secondary metabolites, consequently impacting the medicinal potency and effectiveness of the plants [42, 43]. Prior research has demonstrated that adequate or plentiful phosphorus levels promote the development of root structure, weight, and saponin concentration in plants like *American ginseng*. Additionally, phosphorus greatly augments the alkaloid content in plants such as *Sophora flavescens*, *Lupinus angustifolius, Lolium perenne*, and other species [44–48]. However, in certain soils, although phosphorus content may be rich, a substantial portion exists in forms inaccessible to plants, leading to phosphorus deficiency [49, 50]. Confronted with this limitation (phosphorus deficiency), plants have evolved various developmental, biochemical, and symbiotic adaptation strategies, resulting in the accumulation of specific secondary metabolites [51]. Low phosphorus, for example, makes *Bupleurum chinense* make more saponins, increases the amount of flavonoids (mainly kaempferol and quercetin) in the roots of *Cajanus cajan* and *Pak choi*, and makes *Dendrobium officinale* store more alkaloids and polysaccharides [49, 52, 53]. Consequently, carefully considering and applying phosphorus in the cultivation of medicinal plants can foster robust plant growth and enhance the yield and quality of medicinal herbs.

Finally, potassium, as a key macro-nutrient element, plays a crucial physiological role in plants, participating in key biochemical processes such as regulating osmotic pressure, maintaining ionic homeostasis, sugar transfer, promoting photosynthesis, and "activating" a wide range of enzymes in the plant, which are critical for plant growth, development, and adaptation to the environment [26, 54, 55]. In medicinal plants, an appropriate supply of potassium has a significant regulatory effect on the synthesis and accumulation of secondary metabolites such as alkaloids and anthraquinone compounds [26, 56]. For example, sufficient potassium increases the concentration of alkaloids like vincristine in the roots and leaves of *Catharanthus roseus*, and the content of ephedrine, pseudoephedrine, and flavonoids in *Ephedra sinica* rises with an increase in potassium supply [57, 58]. A severe potassium deficit significantly increases the quantity of alkaloids in the seeds of three sweet cultivars of *Lupinus angustifolius* (*Danja*, *Gungurru*, and *Yorrel*), as observed in a study by Gremigni et al. in 2001 [59]. These findings highlight the significance of potassium in the creation of alkaline compounds. In addition, appropriate potassium levels also enhance the growth and accumulation of secondary metabolites in medicinal plants such as *Fritillaria thunbergii* and *Cannabis sativa* [56, 60, 61]. These research findings underscore the crucial role of optimal potassium application in regulating the secondary metabolites of medicinal plants. Thus, the judicious application of potassium is significantly important for the efficacy and medicinal value of medicinal plants.

Therefore, judiciously managing exogenous nutrients (nitrogen, phosphorus, and potassium) in a scientific and rational manner can boost the concentration of bioactive compounds in medicinal plants, enhancing their medicinal value. Simultaneously, it aids in enhancing both yield and quality, offering substantial support for the sustainable cultivation of medicinal plants. Nonetheless, there is a scarcity of research on the nutrient regulation response in *A. tanguticus*. So, it is very important, both theoretically and practically, to figure. out how nitrogen, phosphorus, and potassium effect on the growth, main alkaloids (anisodine, anisodamine, scopolamine, and atropine) content and alkaloids yield of *A. tanguticus*. To address this research gap, this study hypothesizes that optimizing the individual application rates of nitrogen (N), phosphorus (P), and potassium (K) nutrients can significantly enhance the alkaloid content and overall yield of *A. tanguticus*. The specific objectives are as follows: 1. To determine the optimal nitrogen application rate for maximizing alkaloid content and yield in *A. tanguticus*; 2. To evaluate the optimal phosphorus application rate for enhancing growth and alkaloid accumulation in *A. tanguticus*; 3. To investigate the optimal potassium application rate for increasing alkaloid content accumulation in *A. tanguticus*; 4. To develop an integrated nutrient strategy for N, P, and K to provide theoretical support and practical guidance for the efficient cultivation of *A. tanguticus*.

This research aims to provide critical support for the scientific cultivation and nutrient management of *A. tanguticus*, thereby improving its medicinal value and market competitiveness.

## Materials and methods

### Experimental site

The experimental site is located in Lanlongkou Town, Huangzhong County, Xining City, Qinghai Province, China (101.48° E, 36.76° N), with an average elevation of 2480 m. The annual average sunshine hours, temperature, and precipitation are 2588.3 h, 0 ~ 5 ℃, and 360 ~ 650 mm, respectively. Precipitation is mainly concentrated from July to September, accounting for more than 50% of the annual precipitation. The annual evaporation is 900 ~ 1000 mm, and the frost-free period is 150 ~ 190 days. The physical and chemical properties of the soil in the experimental sample plots are shown in Table 1.

**Table 1:** Soil physical and chemical properties of experimental sample plots

Year	PH	Conductivity	Effective phosphorus (mg/kg)	Total phosphorus (g/kg)	Total nitrogen (%)	Organic matter (g/kg)	K^+^ (mg/kg)
2021	7.35	1116.56	25.62	1.067	0.122	19.68	10,113.07
2022	7.52	465.22	29.73	1.027	0.118	17.86	7636.61

## Plant sources and experimental design

The seedling cultivation experiment of *A. tanguticus* was conducted in 2019 at the Chinese Academy of Sciences Haidong Experimental Station, located in Ledu District, Haidong City, Qinghai Province. After one year of seedling growth, uniform and robust seedlings were excavated in late April 2020 and transplanted to the experimental field in Lanlongkou Town, Huangzhong County, Xining City, Qinghai Province. After transplantation, thorough irrigation was carried out. All samples were identified by Professor Guoying Zhou (Northwest Institute of Plateau Biology, Chinese Academy of Science). The voucher specimens were kept in the Museum of Tibetan Plateau Biology, CAS (HNWP–00018164).

**Fig. 1:**
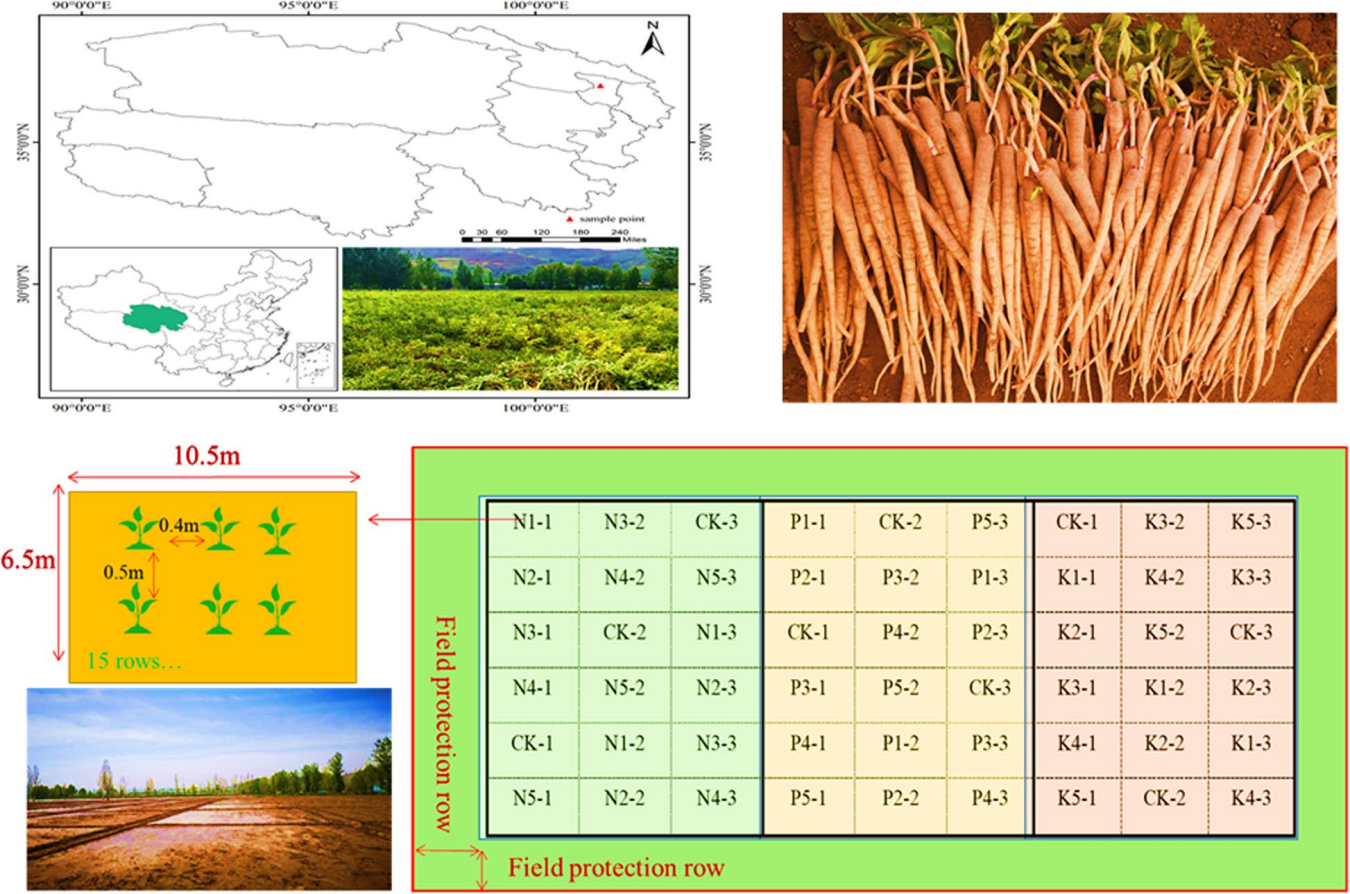
Layout of experimental sample plots

A randomized block experimental design was employed, with nitrogen, phosphorus, and potassium added separately. The specific addition amounts are detailed in Table 2. All additives were applied in a single dose on April 12, 2021, and 2022. Each treatment was repeated three times. Nitrogen was applied using urea (CO(NH_2_)_2_ 46%), phosphorus using calcium superphosphate (CaP_2_H_4_O_8_ 20%), and potassium using potassium chloride (K_2_ O 60%). Each plot measured 10.5 m in length and 6.5 m in width, covering an area of 68.25 m^2^, for a total of 54 plots. The plant spacing was 0.4 m × 0.5 m, with 15 rows per plot and a total of 342 plants per plot. The layout of the experimental sample plots is shown in Fig. 1.

**Table 2:** Levels of nitrogen, phosphorus, and potassium additives(kg/ha)

Nitrogen	CO(NH_2_)_2_	Phosphorus	CaP_2_H_4_O_8_	Potassium	K_2_ O
CK	0	CK	0	CK	0
N1	75	P1	600	K1	75
N2	150	P2	750	K2	112.5
N3	225	P3	900	K3	150
N4	300	P4	1050	K4	187.5
N5	375	P5	1200	K5	225

## Field maintenance

During the initial phase, frequent hand-weeding is carried out on a monthly basis due to the rapid growth of weed roots. Following the sprouting of seedlings, any plants that are affected by disease are swiftly eliminated and disposed of.

## Sampling

Plant samples were collected on June 10th, August 13th, and October 7th in 2021, and on June 12th, August 13th, and October 14th in 2022, labeled as S-Green, S-Growth, S-*Wilting*, T-Green, T-Growth, and T-Wilting, respectively. Each year’s sampling in June, August, and October represents the greening period, growth period, and withering period of *A. tanguticus*, respectively. Five plants were randomly sampled from each plot, and each treatment was replicated three times. After harvesting, the roots were cleaned of soil, and the aboveground parts and roots were weighed for fresh weight. Measurements were taken of pertinent growth markers, including plant height, root length, and root diameter. Afterwards, root slices and aboveground parts were separately placed in mesh bags, air-dried in a well-ventilated environment, and then weighed for dry weight after complete drying. The samples were then ground, sieved through a 60-mesh sieve, and stored in sealed bags for later use.

## Methods for the determination of alkaloids

The reference solution was prepared by weighing 0.0004 g of each standard compound (anisodamine, anisodine, scopolamine, and atropine) and dissolving them in the mobile phase. This resulted in a standard solution with a concentration of 0.4 mg/mL.

To prepare the test samples, precisely measure 2.00 g of *A. tanguticus* (the aboveground sections and roots were measured separately) powder and transfer it into a 150—mL conical flask. Dispense 4 mL of ammonia solution, thoroughly combine, allow to settle for a duration of 10 min, introduce 100 mL of chloroform, and accurately determine the overall mass. Subject the solution to ultrasonication for a duration of 30 min, subsequently allow it to cool down to the ambient temperature, measure its weight, compensate for any weight loss by adding chloroform, and then filter it through defatted cotton. Extract 100 mL of the liquid that has passed through a filter, remove the liquid by evaporation using a device that rotates, dissolve the remaining solid material in 5 mL of methanol, and pass the resulting solution through a 0.45 μm microfiltration membrane into a container for analysis using liquid chromatography (HPLC).

Moisture determination of test samples: Take 2 ~ 5 g of samples from each period of each year and determine the moisture content according to the moisture determination method (General Rule 0832 Second Method) at 105 °C. The results of the moisture determination for *A. tanguticus* samples are shown in Supplementary.

High-performance liquid chromatography (HPLC) parameters: The tropane alkaloid components were determined using a mobile phase (13:87) consisting of acetonitrile and 30 mmol/L potassium dihydrogen phosphate (containing 0.08% triethylamine) with a pH of 6.0 adjusted using phosphoric acid. The analytical column used was an Agilent 5HC-C18, with a fixed column temperature of 35 °C. The injection volume was set at 10 μl, the flow rate at 1 mL/min, and the detection wavelength at 210 nm. During the experiment, the main instruments and reagents involved are detailed in Supplementary Table 2 and Supplementary Table 3, respectively. The stability testing conducted during the experiment is shown in Supplementary Fig. 1. The content was calculated based on the peak area using the external standard method. The formula for calculating the content is as follows:

$$\text{Content}\,\%=\frac{A_{\text{sample}}\times C_{\text{standard}}\times 100\times 5}{A_{\text{standard}}\times m_{\text{sample}}\times1000\times 100\times (1-\text{Moisture}\%)}\times 100\%$$

where *C*_standard_ is the concentration of the reference solution, in mg/mL, *A*_sample_ is the peak area of the sample; *m*_sample_ is the weight of the sample; and *A*_standard_ is the average peak area of the reference solution.

## Statistical analysis

A Data was systematically arranged utilizing Microsoft Excel 2021 (Microsoft, USA), and tables were generated. Data analysis and plotting were performed using R software (R Core Team. R: A Language and Environment for Statistical Computing. R Foundation for Statistical Computing, 2021). Detailed summaries of experimental data, including agronomic traits, biomass, average values, and standard deviations of major alkaloids at different treatments and periods, were obtained using the dplyr package. ANOVA was subsequently performed using the emmeans software to comprehensively examine the interactions between treatments and times, as well as to assess their significant differences. Tukey’s multiple comparison was precisely determined using the multcomp and emmeans packages, and significant differences between different treatment combinations were displayed using letter annotations. To verify the robustness of the differences, t-tests were further conducted to determine statistical significance at *P* < 0.05 (*), *P* < 0.01 (**), and *P* < 0.001 (***). Subsequently, response surface analysis was conducted using the "rsm" and "rgl" programs to derive the response surface model for achieving the highest possible alkaloid yield of *A. tanguticus*. Ultimately, all measurements of indicators were standardized based on the ideal time for harvesting and the specific type of nutrient used. The "lm" function was used to conduct regression analysis, and the "glmnet" package was utilized to execute lasso regression. This was done to acquire the C-D production function for the alkaloid yield of *A. tanguticus*. The software packages utilized in the plotting procedure comprise "ggplot2" for generating bar charts, error bars, and other visualization components; "ggpubr" for incorporating significance markers and labels to ggplot2 charts; "ggsignif" for exhibiting significance markers between two groups in charts; "RColorBrewer" for selecting color schemes for graphics; and "multcompView" for visualizing multiple comparisons.

## Results

### Effects of macro-nutrient exogenous regulation on growth traits of *A. tanguticus*

The impact of nitrogen (N), phosphorus (P), and potassium (K) on the growth traits of *A. tanguticus*, including plant height, root length, and root diameter, revealed significant effects. Nitrogen at different levels exhibited a significant influence on plant height, root length, and root diameter, with the most pronounced effects observed during the growth period. Particularly during the T-Growing period, the N5 level significantly increased plant height, root length, and root diameter by 37.55 cm, 11.92 cm, and 3.01 cm, respectively, representing growth rates of 38.01%, 38.74%, and 40.60%. However, there were no statistically significant differences in the withering period for different nitrogen addition treatments (Table 3). Phosphorus showed a significant increase in plant height, root length, and root diameter. In the S-Growing and T-Growing periods, the P3 level significantly increased plant height by 6.97 cm and 18.09 cm, respectively, compared to CK. In the S-Greening period, the P3 level significantly increased root length by 6.9 cm, while differences in root length between phosphorus levels were not statistically significant in other periods. During the T-Greening and T-Growing periods, root diameter was significantly higher in the P3 and P4 levels compared to CK, with increases of approximately 1.63 cm and 2.84 cm, respectively (Table 3). In terms of potassium treatment, the K1 and K2 levels significantly outperformed other levels in plant height, especially during the S-Growing and T-Growing periods, where the K1 level was 24.14 cm and 26.78 cm higher than K5, respectively. For root length, the K1 and K2 levels in T-Greening significantly exceeded CK, with increases of 5.77 cm and 10.37 cm, respectively, while differences in other periods were not significant. As for root diameter, K2 was significantly higher than K5 in the S-Wilting period, and in the T-Wilting period, K1 was also significantly higher than K5. The results suggest that the K1 and K2 levels promote the growth of *A. tanguticus*, particularly in plant height and root diameter, showing significant effects. Overall, the impact of nutrient types on plant growth traits is significant, with nitrogen and potassium fertilizers showing significance across multiple indicators (Table 3).

**Table 3:** Effects of macro-nutrient exogenous regulation on plant height, root length and root diameter at different growth stages of *A. tanguticus*(cm)

Ind	Treatments	Period					
	Mean ± SD *n* = 15	S-Greening	S-Growing	S-Wilting	T-Greening	T-Growing	T-Wilting
Plant Height	CK	60.88 ± 14.54b	143.50 ± 16.37b	129.87 ± 14.83a	86.03 ± 9.20c	98.79 ± 15.32c	101.03 ± 17.12a
	N1	64.07 ± 9.21ab	146.80 ± 18.56ab	123.92 ± 21.67a	87.53 ± 7.73bc	120.14 ± 17.6bc	105.07 ± 22.92a
	N2	62.87 ± 13.66ab	145.27 ± 10.6ab	132.73 ± 12.81a	90.20 ± 8.27abc	107.66 ± 14.41b	95.83 ± 15.92a
	N3	67.07 ± 10.28ab	149.86 ± 13.61ab	136.73 ± 14.82a	98.12 ± 6.74ab	120.28 ± 13.65b	102.59 ± 7.47a
	N4	75.27 ± 9.48ab	152.47 ± 11.72ab	137.93 ± 8.22a	98.73 ± 7.73ab	118.91 ± 8.48b	104.20 ± 10.89a
	N5	68.40 ± 7.52a	158.67 ± 11.59a	138.00 ± 13.85a	102.20 ± 5.54a	136.34 ± 11.23a	108.53 ± 10.03a
	CK	60.88 ± 14.54a	143.50 ± 16.37abc	129.87 ± 14.83ab	86.03 ± 9.2ab	98.79 ± 15.32c	101.03 ± 17.12ab
	K1	60.20 ± 5.76a	158.27 ± 14.47a	133.67 ± 25.87ab	91.80 ± 6.06ab	131.57 ± 14.88a	107.18 ± 16.48a
	K2	61.93 ± 18.57a	154.73 ± 11.92ab	142.13 ± 20.15a	96.40 ± 8.77a	125.43 ± 11.43a	114.25 ± 14.79ab
	K3	53.53 ± 21.64a	137.53 ± 24.44bc	133.33 ± 33.35ab	85.76 ± 14.41ab	105.81 ± 22.01bc	91.92 ± 18.94b
	K4	49.67 ± 16.25a	133.00 ± 37.19c	117.87 ± 31.29b	86.13 ± 12.15ab	120.07 ± 20.22ab	91.64 ± 24.8b
	K5	49.07 ± 18.81a	134.13 ± 30.09c	125.07 ± 18.55ab	75.00 ± 15.49b	104.79 ± 12.08bc	92.56 ± 13.98b
	CK	60.88 ± 14.54b	143.50 ± 16.37b	129.87 ± 14.83a	86.03 ± 9.20a	98.79 ± 15.32b	101.03 ± 17.12a
	P1	56.67 ± 11.90a	138.73 ± 17.44ab	130.14 ± 15.82a	85.73 ± 10.35a	124.16 ± 14.39a	89.51 ± 18.37a
	P2	58.40 ± 11.30a	146.67 ± 25.13b	130.53 ± 19.62a	86.00 ± 8.01a	108.82 ± 8.07ab	95.93 ± 16.93a
	P3	59.53 ± 12.82a	150.47 ± 14.53a	130.53 ± 8.78a	92.14 ± 7.69a	116.88 ± 9.91a	94.73 ± 20.14a
	P4	67.40 ± 9.03a	145.87 ± 16.8b	128.67 ± 11.97a	83.47 ± 6.22a	112.47 ± 17.47ab	89.88 ± 15.13a
	P5	61.47 ± 10.25a	147.13 ± 14.67b	139.53 ± 16.79a	83.72 ± 14.41a	123.31 ± 15.78a	97.86 ± 16.99a
Root Length	CK	31.26 ± 6.36a	31.19 ± 5.74a	41.22 ± 7.05a	28.04 ± 6.51b	30.78 ± 8.02c	40.08 ± 7.26a
	N1	31.07 ± 5.52a	30.33 ± 5.47a	43.08 ± 6.36a	31.93 ± 7.27ab	35.67 ± 3.08bc	42.27 ± 4.40a
	N2	34.07 ± 4.77a	31.67 ± 6.79a	40.33 ± 6.29a	29.33 ± 5.79ab	35.93 ± 3.68bc	39.48 ± 7.04a
	N3	32.60 ± 5.74a	26.57 ± 3.8a	40.53 ± 5.04a	31.65 ± 6.93ab	36.45 ± 3.89abc	40.00 ± 6.36a
	N4	31.67 ± 6.52a	27.67 ± 3.7a	43.71 ± 7.36a	31.33 ± 3.87ab	38.14 ± 3.28ab	38.20 ± 5.00a
	N5	30.93 ± 4.83a	29.47 ± 9.16a	41.79 ± 3.42a	35.00 ± 5.14a	42.70 ± 8.46a	42.73 ± 4.99a
	CK	31.26 ± 6.36a	31.19 ± 5.74a	41.22 ± 7.05a	28.04 ± 6.51ab	30.78 ± 8.02a	40.08 ± 7.26a
	K1	34.67 ± 4.24a	32.40 ± 6.76a	41.33 ± 6.14a	33.00 ± 7.46a	35.45 ± 6.30a	42.97 ± 8.58a
	K2	36.73 ± 6.18a	32.07 ± 5.18a	40.93 ± 6.24a	31.67 ± 7.75a	34.41 ± 6.19a	42.31 ± 6.01a
	K3	31.00 ± 5.59a	34.13 ± 5.48a	43.53 ± 10.10a	30.65 ± 8.14a	29.05 ± 8.60a	39.28 ± 4.66a
	K4	34.20 ± 5.92a	33.2 ± 5.33a	38.13 ± 5.44a	29.20 ± 5.56ab	34.95 ± 5.10a	39.99 ± 5.97a
	K5	31.57 ± 4.93a	31.47 ± 4.39a	41.00 ± 7.87a	22.71 ± 6.19b	33.15 ± 7.43a	38.33 ± 5.64a
	CK	31.26 ± 6.36b	31.19 ± 5.74a	41.22 ± 7.05a	28.04 ± 6.51a	30.78 ± 8.02a	40.08 ± 7.26a
	P1	31.87 ± 7.85ab	28.13 ± 5.71a	39.79 ± 5.62a	26.13 ± 6.89a	34.64 ± 5.19a	37.15 ± 4.90a
	P2	35.80 ± 6.76ab	27.87 ± 4.00a	44.33 ± 5.25a	28.80 ± 5.81a	30.78 ± 10.68a	37.61 ± 6.20a
	P3	38.20 ± 6.21a	27.67 ± 5.70a	40.87 ± 7.37a	25.79 ± 4.19a	33.57 ± 5.15a	36.87 ± 6.12a
	P4	33.40 ± 6.21ab	30.8 ± 4.33a	40.47 ± 3.44a	30.13 ± 4.02a	33.98 ± 4.98a	37.44 ± 5.20a
	P5	32.53 ± 5.64ab	32.13 ± 2.9a	39.47 ± 4.64a	26.56 ± 5.18a	33.99 ± 5.25a	35.93 ± 6.04a
Root Diameter	CK	4.15 ± 1.21a	6.19 ± 1.37a	7.86 ± 2.01a	6.85 ± 1.28b	7.41 ± 1.54c	7.95 ± 1.78a
	N1	4.51 ± 0.83a	6.29 ± 1.22a	7.91 ± 1.63a	6.16 ± 1.39b	8.34 ± 1.79	8.87 ± 1.59a
	N2	4.62 ± 1.65a	6.75 ± 1.51a	7.89 ± 2.15a	7.16 ± 1.90b	8.08 ± 1.55bcd	8.48 ± 1.72a
	N3	4.95 ± 1.55a	5.77 ± 0.96a	7.65 ± 1.39a	7.69 ± 1.23ab	10.03 ± 2.72ab	7.92 ± 2.00a
	N4	4.31 ± 1.13a	5.34 ± 1.19a	8.37 ± 2.39a	7.87 ± 2.33ab	9.21 ± 2.47abc	7.33 ± 1.41a
	N5	3.99 ± 1.16a	5.70 ± 1.07a	7.49 ± 1.78a	9.03 ± 2.18a	10.42 ± 2.67a	8.27 ± 1.88a
	CK	4.15 ± 1.21a	6.19 ± 1.37a	7.86 ± 2.01ab	6.85 ± 1.28b	7.41 ± 1.54a	7.95 ± 1.78ab
	K1	4.75 ± 1.46a	6.12 ± 1.87a	8.01 ± 1.92a	7.38 ± 1.51ab	7.55 ± 1.39a	9.26 ± 1.98a
	K2	5.26 ± 1.78a	5.88 ± 1.40a	6.90 ± 1.15a	8.11 ± 1.58a	8.61 ± 2.66a	8.00 ± 2.10ab
	K3	3.38 ± 0.99a	5.99 ± 1.43a	8.02 ± 2.28ab	6.47 ± 2.42ab	7.83 ± 3.55a	7.51 ± 2.60ab
	K4	3.77 ± 1.59a	5.51 ± 1.31a	6.78 ± 2.22ab	7.02 ± 2.97ab	7.75 ± 1.55a	8.36 ± 1.98ab
	K5	4.44 ± 1.68a	5.62 ± 1.25a	6.80 ± 1.82b	5.77 ± 1.80ab	6.81 ± 1.89b	6.99 ± 1.11b
	CK	4.15 ± 1.21a	6.19 ± 1.37ab	7.86 ± 2.01a	6.85 ± 1.28b	7.41 ± 1.54b	7.95 ± 1.78a
	P1	4.50 ± 1.11a	7.29 ± 1.70a	8.23 ± 1.17a	7.35 ± 2.24ab	8.2 ± 2.15b	8.92 ± 1.68a
	P2	4.98 ± 1.51a	5.97 ± 0.77ab	9.02 ± 1.51a	8.23 ± 2.64ab	8.69 ± 1.20ab	8.73 ± 2.74a
	P3	4.93 ± 1.22a	5.54 ± 1.61b	7.81 ± 1.36a	8.48 ± 1.37a	8.63 ± 1.11ab	8.62 ± 2.13a
	P4	4.93 ± 1.04a	5.91 ± 1.16ab	7.79 ± 1.48a	7.87 ± 1.73ab	10.25 ± 2.22a	8.29 ± 2.08a
	P5	4.59 ± 1.50a	6.20 ± 1.42ab	8.69 ± 1.61a	7.16 ± 1.83ab	8.49 ± 1.76b	7.18 ± 1.97a

Tukey’s (HSD) test indicates that the column means displaying distinct letters are statistically significant (*p-value* < 0.05)

It is noteworthy that the plant height, root length, and root diameter of *A. tanguticus* exhibited statistically significant differences among years, growth stages, and different treatments. Additionally, the interactions between years and nutrient addition, growth stages, and nutrient addition were statistically significant (*P* < 0.001) (Table 4). The interactions between plant height and root diameter and between years and nutrient addition, as well as between growth stages and nutrient addition, were not statistically significant. In addition, the impact effects of phosphorus and potassium additions on plant height and root length were also not statistically significant (Table 4).

**Table 4:** Statistical analysis of traits of *A. tanguticus*(cm)

	Plant height		Root length		Root diameter	
	*p-value*	F	*p-value*	F	*p-value*	F
Year (2021:2022)	< 0.001	325.822	< 0.05	4.845	< 0.001	544.76
Period	< 0.001	1593.427	< 0.001	275.31	< 0.001	199.026
Type (N: P: K)	< 0.001	14.745	< 0.01	5.175	< 0.001	11.744
Treatment	< 0.001	13.107	< 0.001	3.068	< 0.001	4.559
Year*Treatment	< 0.01	2.272	< 0.001	2.541	< 0.001	3.744
Treatment*Period	< 0.001	2.503	< 0.001	1.782	< 0.001	1.789
Year: Type	ns	1.221	< 0.05	10.657	ns	1.365
Type: Period	ns	1.251	< 0.001	2.212	ns	1.448
N Vs P	< 0.05	2.4671	< 0.05	2.463	ns	-0.9436
N Vs K	< 0.05	2.148	ns	0.511	< 0.01	2.73235
P Vs K	ns	-0.188	ns	-1.913	< 0.001	3.75611

*ns* non-significant

## Effects of macro-nutrient exogenous regulation on biomass accumulation of *A. tanguticus*

**Fig. 2:**
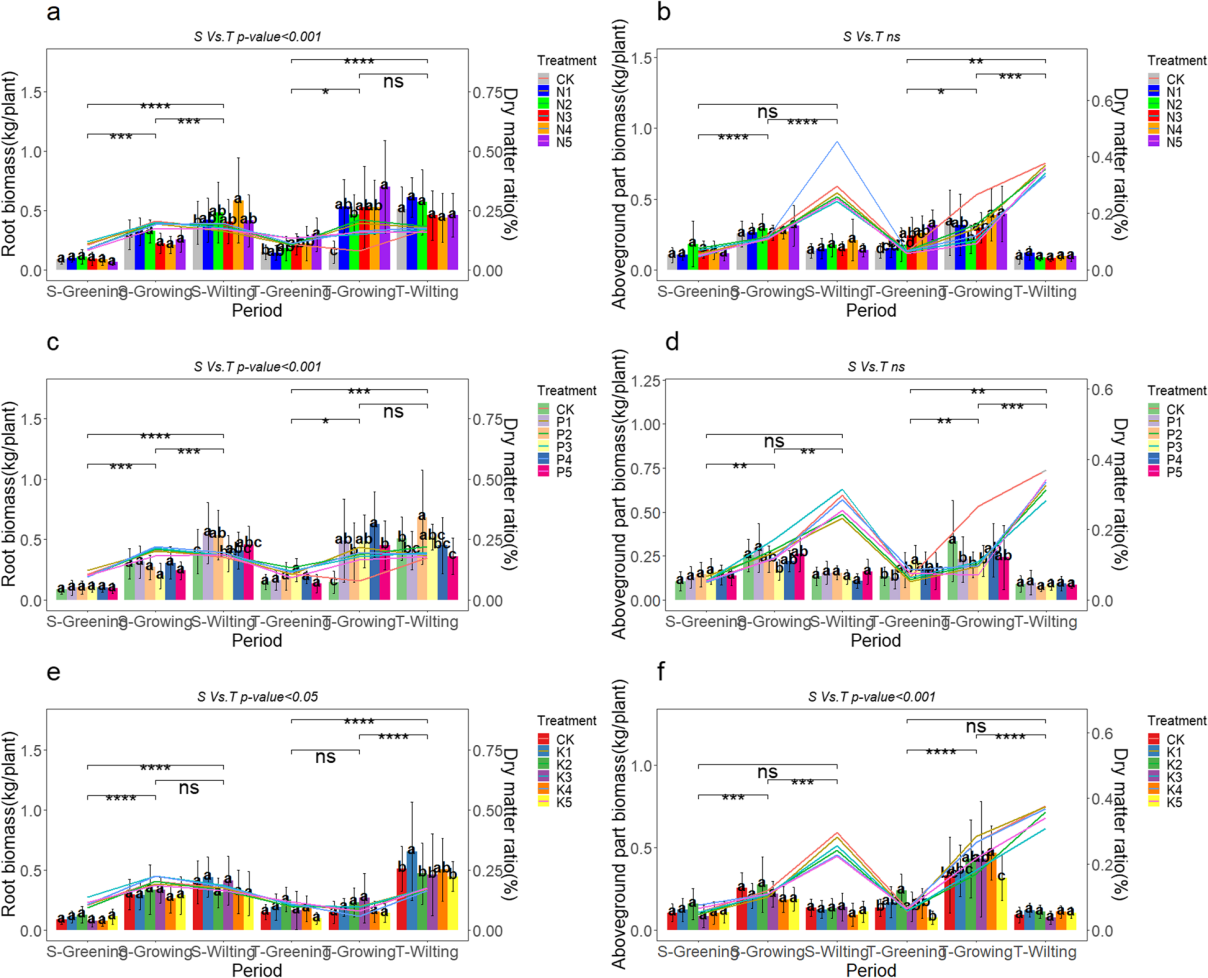
Biomass and dry matter ratio of roots and aboveground parts of *A. tanguticus* under different nutrient additions (S vs. T = 2021 vs. 2022, Tukey’s (HSD) test indicates statistical significance marked by letter symbols (*p-value* < 0.05), while the T-test identifies significant differences at the levels of *P* < 0.05 (*), *P* < 0.01 (**), and *P* < 0.001 (***), with ‘ns’ indicating no significant difference. a, c, and e indicate the effects of N, P, and K on the root biomass and dry matter rate of *A. tanguticus*, respectively; b, d, and f indicate the effects of N, P, and K on the biomass and dry matter rate of the aboveground parts of *A. tanguticus*, respectively.)

Nitrogen, phosphorus, and potassium additions all had significant impacts on the biomass accumulation of *A. tanguticus*. Our results indicate that under nitrogen addition, the effects varied between different nitrogen levels and growth stages but generally promoted biomass accumulation, as illustrated in Fig. 2a and 2b. Specifically, the N5 level significantly affected both root and aboveground biomass of *A. tanguticus*, with the highest increases observed during the T-Greening and T-Growing periods. The root biomass increased by 98.22% and 377.81%, reaching 1.98 times and 4.78 times that of CK, respectively, while the aboveground biomass increased by 2.35 times and 1.19 times that of CK. Other nitrogen levels also showed inconsistent effects between different growth stages but generally promoted biomass accumulation. The dry weight ratio of the roots was 2.20 times greater during the T-Growing phase when nitrogen was added (Fig. 2a). However, the dry weight ratio of the aboveground parts was generally somewhat lower than the control group under high nitrogen concentrations (Fig. 2b). The root-to-shoot ratio of *A. tanguticus* showed that low nitrogen levels (N1, N2) generally exceeded high nitrogen levels (N4, N5) (Supplementary Fig. 2a). The addition of phosphorus had a considerable impact on root biomass, with the P2 level causing a rise of 0.137 kg and 0.177 kg during the S-Wilting and T-Wilting periods, respectively. This represents an almost 35% increase in root biomass (Fig. 2c). The impact of phosphorus on root biomass was statistically significant in all growth phases, except for the period between T-Growing and T-Wilting (*P* < 0.01) (Fig. 2c). Meanwhile, there were no notable disparities in the dry weight of the roots. During the T-Greening period, the aboveground biomass of *A. tanguticus* was substantially larger at the P3 level, reaching 1.57 times the biomass of CK. Notably, the ratio of aboveground dry weight for CK (13.12%) was higher than that of the P3 level (12.85%). Additionally, the CK level was the highest during both the T-Growing and T-*Wilting* periods. The overall effect of phosphorus addition on the aboveground biomass of *A. tanguticus* was relatively weak (Fig. 2d). The root-to-shoot ratio of *A. tanguticus* indicated that, when phosphorus was added, lower concentrations (P1, P2) outperformed higher concentrations (P5) during the S-Wilting and T-Wilting periods (Supplementary Fig. 2b). The addition of potassium, specifically at the K1 level, had a considerable impact on the root biomass during the T-Wilting phase. It resulted in a 0.145 kg increase, which represents a 28.34% growth. During other growth stages, the differences between potassium levels were not significant. Overall, the root biomass increased by 0.040 kg to 0.103 kg under the K2 level, with growth rates ranging from 10.84% to 83.5%, and there were no significant differences in root dry weight ratio (Fig. 2e). During the T-Greening and T-Growing periods, the aboveground biomass increased by 76.64% and 29.34%, respectively, under the K2 level. Additionally, the aboveground dry weight ratio was lower under low potassium levels (K1, K2) compared to high potassium levels (K3, K4) during the S-Greening and T-Greening periods (Fig. 2f). The root-to-shoot ratio of *A. tanguticus* showed that the K3 level was the highest during the T-Growing and T-Wilting periods (Supplementary Fig. 2c).

To summarize, the addition of nitrogen had a considerable impact on the accumulation of biomass in *A. tanguticus*, especially in terms of root biomass. The impacts of adding phosphorus and potassium were relatively weaker, particularly in terms of aboveground biomass where the addition of phosphorus did not provide a distinct advantage (Supplementary Fig. 3d, e, and f). Additionally, root dry weight ratio and aboveground dry weight ratio under nitrogen, phosphorus, and potassium additions were higher in the greening and growing periods than in the withering period, with root dry weight ratio being lower than aboveground dry weight ratio in the withering period.

During the T-Growing stage, the addition of potassium resulted in a reduced ratio of root dry weight compared to aboveground dry weight (Fig. 2). The root-to-shoot ratio exhibited a steady trend that aligned with the aboveground dry weight ratio, as shown in Supplementary Fig. 2a, b, and c and Fig. 2b, d, and f. The root-to-shoot ratio changes indicated that the addition of nitrogen, phosphorus, and potassium reached their highest levels during the T-Wilting stage, with phosphorus having the greatest effect, followed by nitrogen and then potassium (Supplementary Fig. 3c, f). From Fig. 2 and Supplementary Fig. 3, it can also be observed that although root biomass and leaf biomass appear to be significantly influenced by potassium and nitrogen levels respectively, root-to-shoot ratio seems to be more affected by phosphorus levels (with a larger dose leading to a greater negative impact). In addition, the root and aboveground biomass, dry weight ratio, and root-to-shoot ratio of *A. tanguticus* showed statistical significance and significant differences between years, growth stages, nutrient types, and their interactions (*P* < 0.001) (Table 5).

**Table 5:** Statistical analysis of root and aboveground part biomass and root-to-Shoot Ratio of *A. tanguticus*(cm)

	Root biomass		Aboveground biomass		Root-to-Shoot ratio	
	*p-value*	F	*p-value*	F	*p-value*	F
Year (2021:2022)	< 0.001	124.34	< 0.001	45.27	< 0.001	246.93
Period	< 0.001	376.02	< 0.001	374.59	< 0.001	321.70
Type (N: P: K)	< 0.001	17.17	< 0.001	5.18	< 0.001	193.58
Treatment	< 0.001	5.18	< 0.001	4.46	< 0.001	2.64
Year*Treatment	< 0.001	3.67	< 0.001	3.56	< 0.05	1.86
Treatment*Period	< 0.001	4.62	< 0.001	2.39	< 0.001	3.18
Year: Type	< 0.001	8.59	< 0.001	55.97	< 0.001	26.90
Type: Period	< 0.001	14.30	< 0.001	95.02	< 0.001	53.91

*ns* = non-significant

## Effects of exogenous macronutrient regulation on the major alkaloids content of *A. tanguticus* roots

**Fig. 3:**
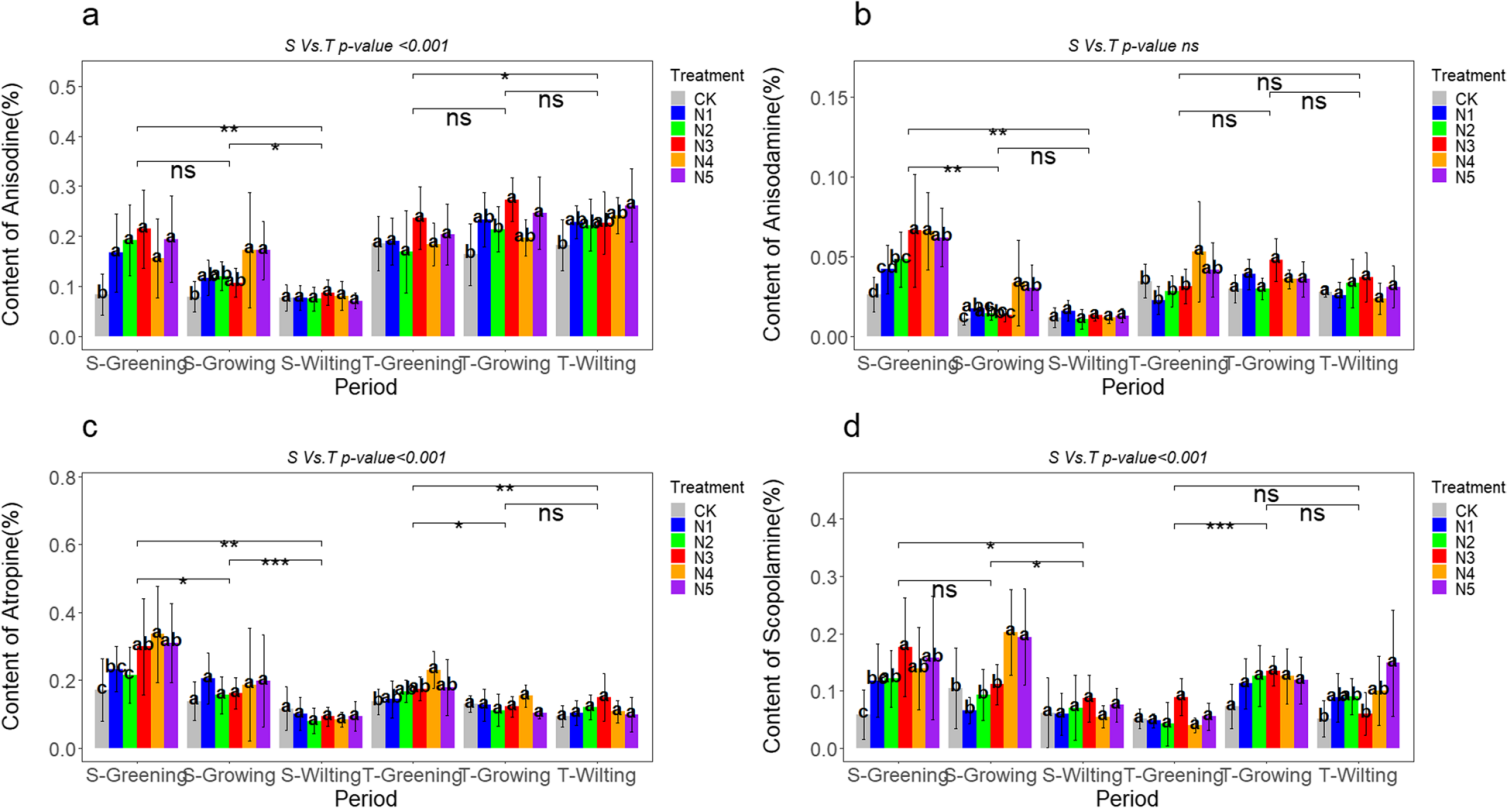
Effect of nitrogen addition on the content of major alkaloids of *A. tanguticus* roots at different stages of growth (S vs. T = 2021 vs. 2022, Tukey’s (HSD) test indicates statistical significance marked by letter symbols (*p-value* < 0.05), while the T-test identifies significant differences at the levels of *P* < 0.05 (*), *P* < 0.01 (**), and *P* < 0.001 (***), with ‘ns’ indicating no significant difference)

**Fig. 4:**
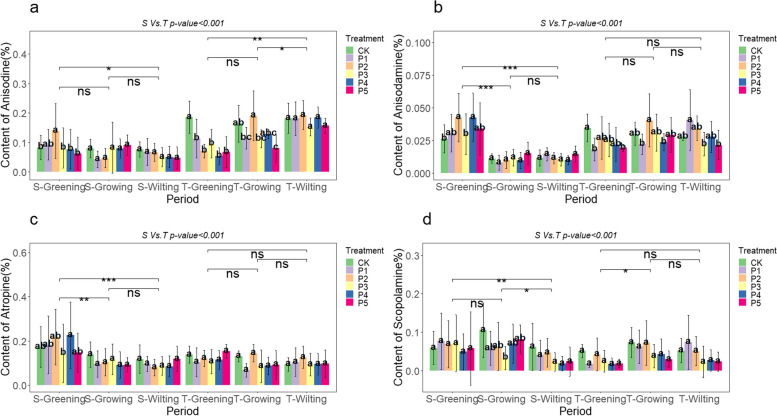
Effect of phosphorus addition on the content of major alkaloids of *A. tanguticus* roots at different stages of growth (Significance level as above)

**Fig. 5:**
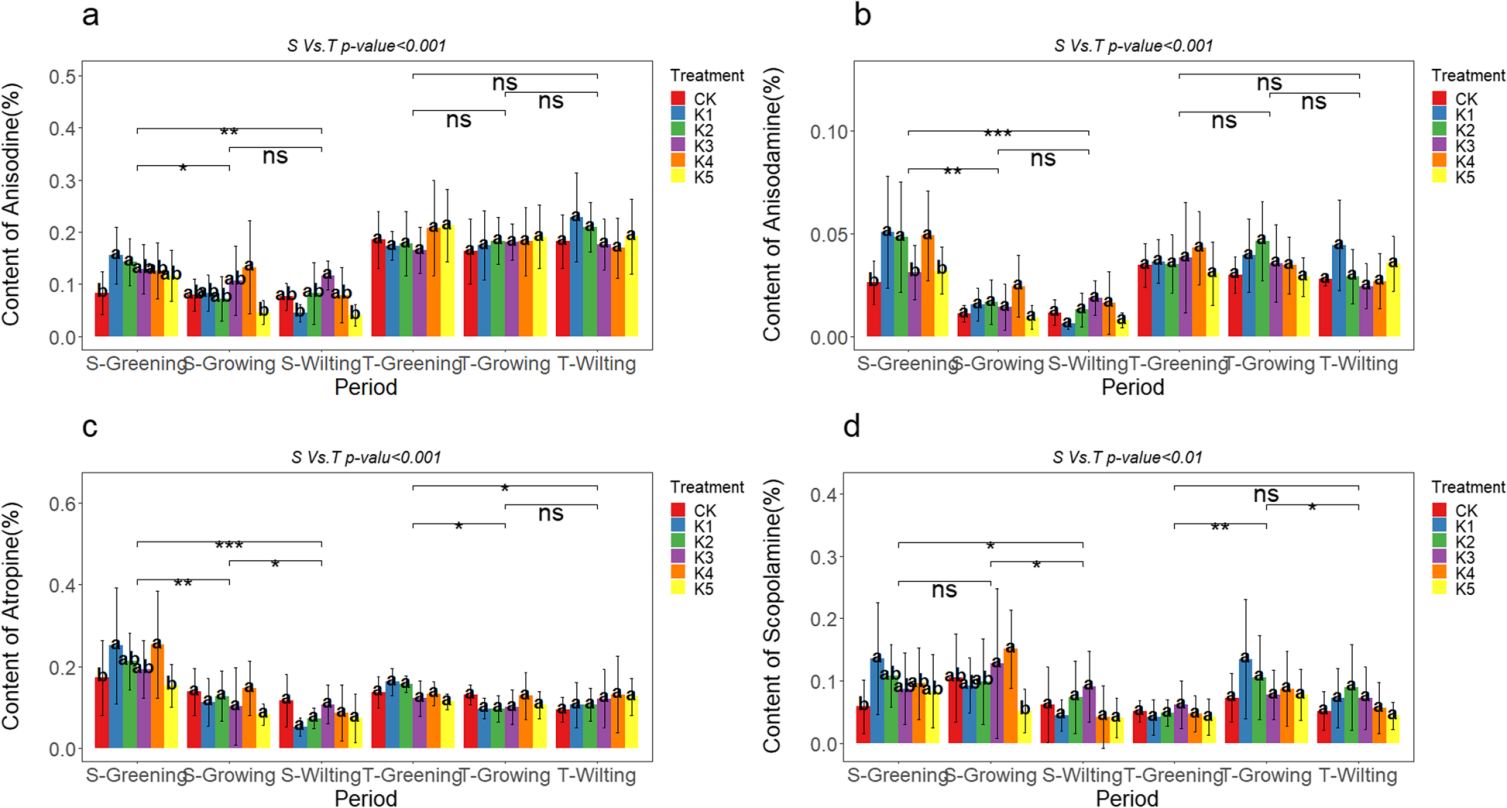
Effect of potassium addition on the content of major alkaloids of *A. tanguticus* roots at different stages of growth (Significance level as above)

The content of anisodine, anisodamine, atropine, and scopolamine in the roots of *A. tanguticus* showed different regulatory patterns in response to the addition of nitrogen, phosphorus, and potassium. The results indicate significant variations in the impact of nitrogen addition on the alkaloid content in the roots of *A. tanguticus* at different growth stages. Specifically, during the S-Greening period, the content of anisodine, anisodamine, atropine, and scopolamine significantly increased by 158.77%, 152.34%, 74.30%, and 200.50%, respectively, under the N3 level. At the N4 level, anisodamine and atropine content significantly increased, reaching 1.54 times and 1.68 times that of CK. Nitrogen addition led to varying degrees of accumulation of these alkaloids (Fig. 3a, b, c, and d). During the S-Growing period, anisodine, anisodamine, and scopolamine content at N4 and N5 levels were significantly higher than CK, increasing by approximately 116.79%, 188.19%, and 89.39%, respectively. Moreover, during the T-Wilting period, scopolamine and hyoscyamine content significantly increased under the N5 level, reaching 0.26% and 0.14%, respectively, representing a 43.83% and 187.28% increase. The addition of phosphorus had various effects on the alkaloid concentration in the roots of *A. tanguticus* at different stages of growth. During the S-Greening period, anisodine and anisodamine content significantly increased at the P2 level, being 1.68 times and 1.62 times that of CK (Fig. 4a, b). Additionally, the content of atropine increased by approximately 31.00% at the P4 level (Fig. 4c). During the T-Growing period, anisodine and anisodamine content significantly increased by 16.48% and 34.60% at the P2 level. Nevertheless, the addition of phosphorus did not have a significant effect on the scopolamine level. In fact, the scopolamine concentration decreased compared to the control group during the S-Growing, S-Wilting, T-Greening, and S-Growing periods (Fig. 4d). Potassium addition significantly influenced the alkaloid content in the roots of *A. tanguticus* at different growth stages. During the S-Greening period, the K1 level significantly affected the content of anisodine, anisodamine, atropine, and scopolamine, increasing by 87.18%, 93.06%, 45.71%, and 130.76%, respectively (Fig. 5a, b, c, d). In addition, the anisodine content increased by 1.67 times at the K4 level during the S-Growing period and 1.52 times during the S-Wilting period. Anisodamine and scopolamine content at the T-Growing and T-Wilting periods showed an increasing trend under low potassium levels (K1, K2) compared to CK. During the T-Wilting period, there was a noticeable upward tendency in the levels of atropine as the potassium concentration increased. However, this trend did not reach a statistically significant level, as shown in Fig. 5c.

**Fig. 6:**
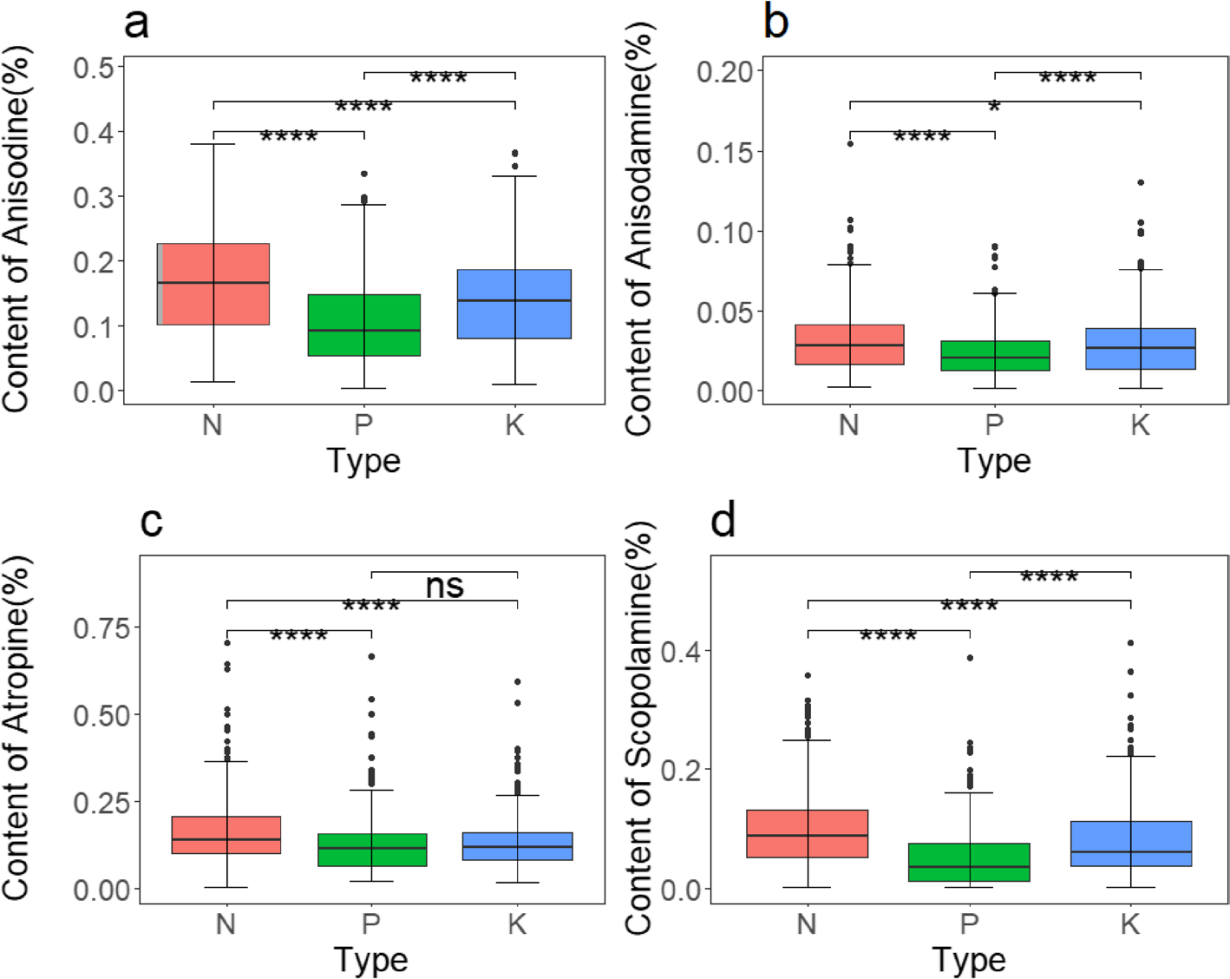
Major alkaloid content of *A. tanguticus* roots in response to different nutrients (t-tests to determine significant differences at the *P* < 0.05 (*), *P* < 0.01 (**), and *P* < 0.001 (***) levels, with "ns" denoting non-significant differences)

To summarize, the addition of nitrogen had a greater and more noticeable enhancing influence on the concentration of these primary alkaloids in the roots of *A. tanguticus*. This effect was followed by the addition of potassium, while the impact of the phosphorus addition was less obvious. Additionally, the content of scopolamine decreased during specific periods. The specific nutritional effects were observed as follows: anisodine, anisodamine, and scopolamine showed a pattern of N > K > P, while atropine exhibited a pattern of N > K ≈ P (Fig. 6a, b, c, d). Furthermore, atropine, anisodamine, anisodine, and scopolamine demonstrated significant statistical differences in years, growth stages, nutrient types, and their interactions (*P* < 0.001), including interactions between different treatments and growth stages (Fig. 3, 4, 5, and Table 6).

**Table 6:** Statistical analysis of the main alkaloid contents of *A. tanguticus* roots(cm)

	Anisodine		Anisodamine		Atropine		Scopolamine	
	*p-value*	F	*p-value*	F	*p-value*	F	*p-value*	F
Year(2021:2022)	< 0.001	579.881	< 0.001	59.803	< 0.001	65.977	< 0.001	43.350
Period	< 0.001	42.240	< 0.001	162.363	< 0.001	90.620	< 0.001	34.880
Type (N:P: K)	< 0.001	134.924	< 0.001	33.690	< 0.001	32.550	< 0.001	78.830
Treatment	< 0.001	7.097	< 0.001	6.864	< 0.001	3.418	< 0.001	6.100
Year*Treatment	< 0.01	2.217	< 0.05	1.936	ns	1.006	< 0.001	0.749
Treatment*Period	< 0.01	2.401	< 0.001	2.661	< 0.01	1.578	ns	1.949
Year: Type	< 0.01	6.290	< 0.01	6.512	< 0.05	3.985	ns	0.690
Type: Period	< 0.001	5.680	< 0.001	4.436	< 0.001	3.564	ns	1.678

*ns* non-significant

## Effect of macro-nutrient exogenous regulation on the alkaloid content in *A. tanguticus* aboveground parts

**Fig. 7:**
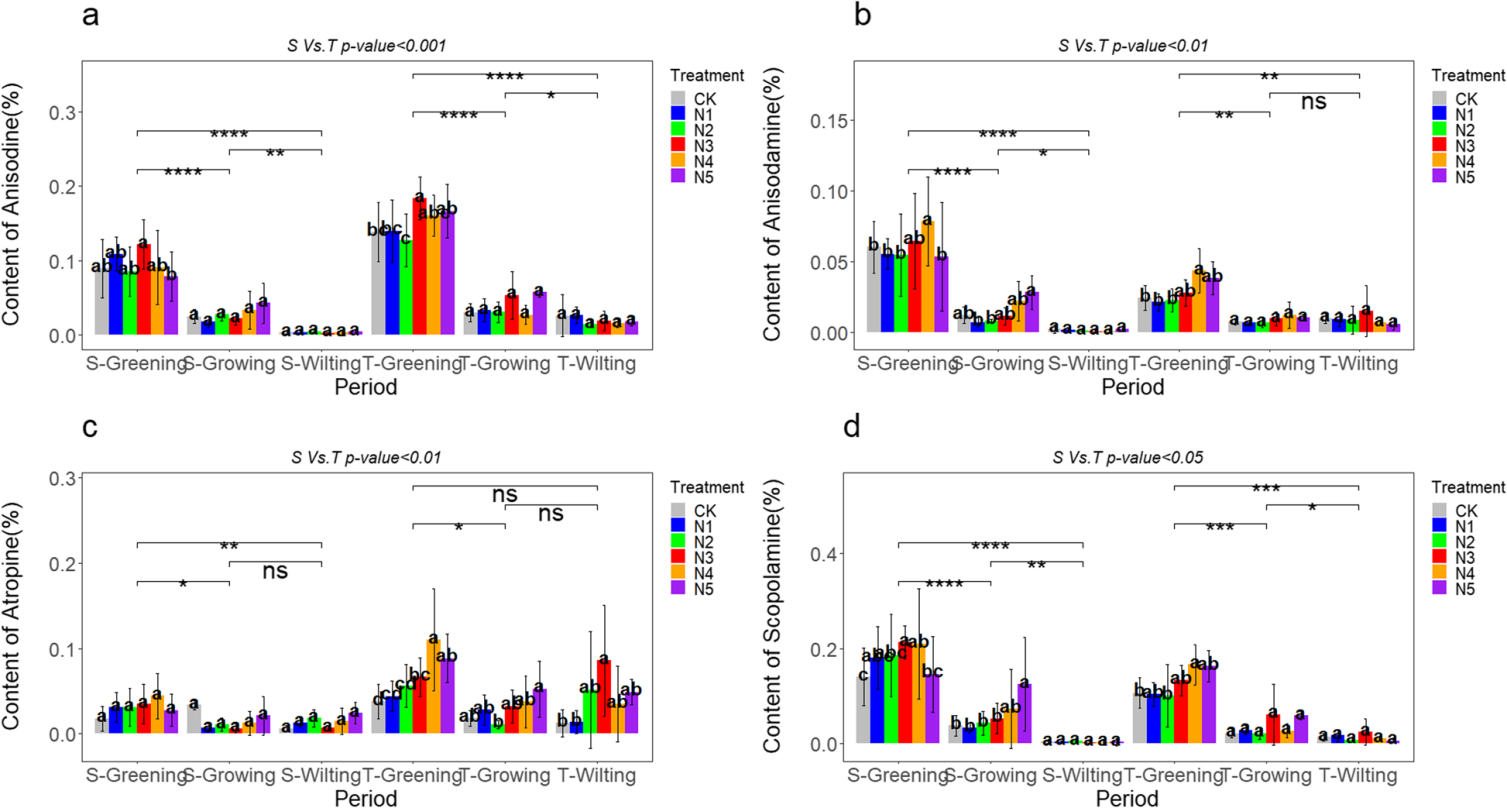
Effect of nitrogen addition on the content of major alkaloids in the aboveground parts of *A. tanguticus* roots at different stages of growth (S vs. T = 2021 vs 2022, Tukey’s (HSD) test indicates statistical significance marked by letter symbols (*p-value* < 0.05), while the T-test identifies significant differences at the levels of *P* < 0.05 (*), *P* < 0.01 (**), and *P* < 0.001 (***), with ‘ns’ indicating no significant difference)

**Fig. 8:**
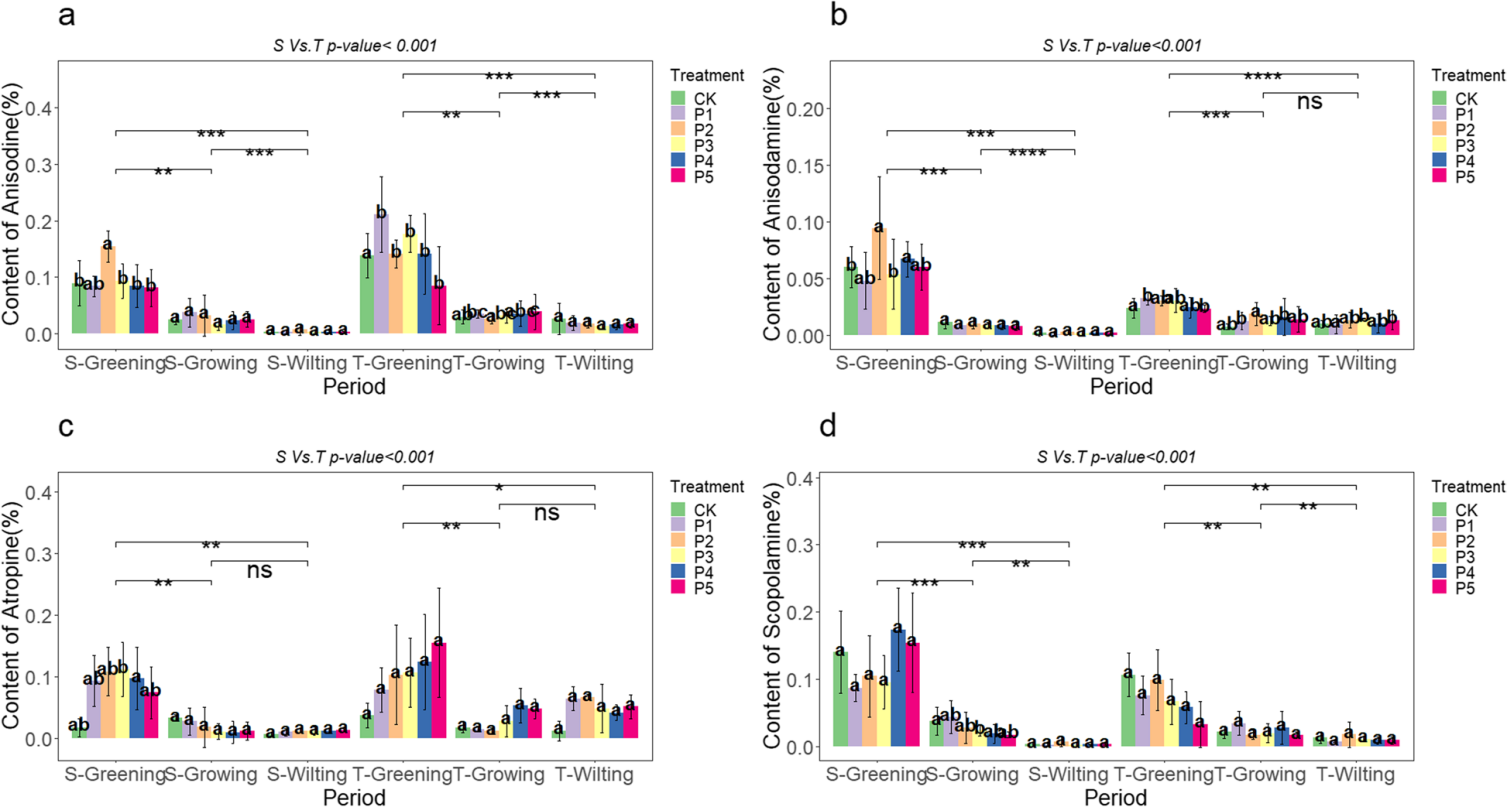
Effect of phosphorus addition on the content of major alkaloids in the aboveground parts of *A. tanguticus* roots at different stages of growth (Significance level as above)

**Fig. 9:**
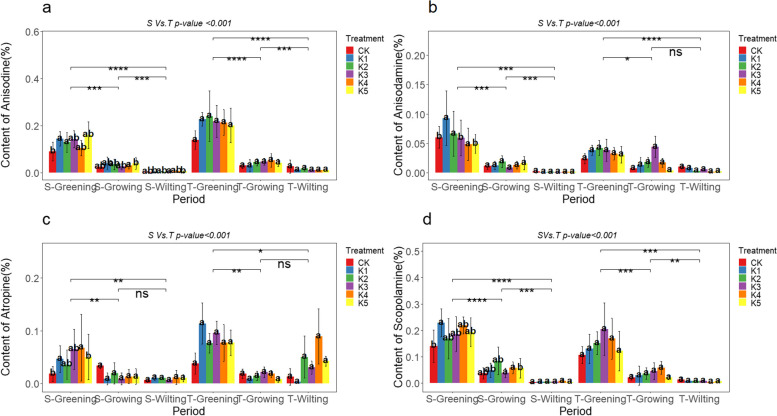
Effect of potassium addition on the content of major alkaloids in the aboveground parts of *A. tanguticus* roots at different stages of growth (Significance level as above)

The content of anisodine, anisodamine, atropine, and scopolamine in the aboveground parts of *A. tanguticus* showed significant regulatory effects under the addition of nitrogen, phosphorus, and potassium nutrients. Our research results revealed that nitrogen addition generally demonstrated a strong promoting effect on *A. tanguticus* during the greening and growing periods. The content of both anisodine and scopolamine increased significantly during the S-Greening and T-Greening periods, with anisodine increasing by 36.66% and 32.92% at the N3 level and scopolamine increasing by 30.35% and 79.01% at the N4 level, respectively (Fig. 7a, b). The concentration of atropine significantly increased at N4 and N5 levels, especially during the T-Greening period, with a notable rise of 191.45% at the N4 level (Fig. 7c). Scopolamine content significantly increased by 51.95% at the N3 level during the S-Greening period, 55.49% at the N4 level during the T-Greening period, and 233.61% at the N5 level during the S-Growing period (Fig. 7d). Phosphorus addition also showed significant effects on the aboveground content of anisodine, anisodamine, atropine, and scopolamine in *A. tanguticus*. Specifically, during the S-Greening period, the P2 level significantly increased the content of anisodine and anisodamine by 73.36% and 56.64%, respectively. During the T-Greening period, the P1 level significantly increased the content of anisodine by 52.62% and anisodamine by 35.36% (Fig. 8a, b). Notably, atropine content exhibited a substantial increase at the P3 level during the S-Greening period and the P5 level during the T-Greening period, with increases of 518.66% and 311.73%, respectively. Different phosphorus levels promoted the generation of aboveground atropine (Fig. 8c). Additionally, except during the S-Greening period, the overall difference in scopolamine was not significant, and the scopolamine content showed a decreasing trend with the increase in phosphorus concentration (Fig. 8d). Potassium addition led to an increase in the content of major alkaloids in the aboveground parts of *A. tanguticus* during the greening period. During the S-Greening period, the K1 level significantly increased the content of anisodine, anisodamine, atropine, and scopolamine by 60.65%, 54.28%, 155.66%, and 62.96%, respectively, as depicted in Fig. 9s a, b, c, and d. Significantly, atropine had the most substantial rise, reaching a remarkable 200.89% at the K1 level throughout the T-Greening period (Fig. 9c). Additionally, anisodine and scopolamine content increased at the K5 level during the S-Growing period by 59.42% and 50.27%, respectively, and at K4 level during the T-Growing period by 78.60% and 170.55%, respectively (Fig. 9a, d).

**Fig. 10:**
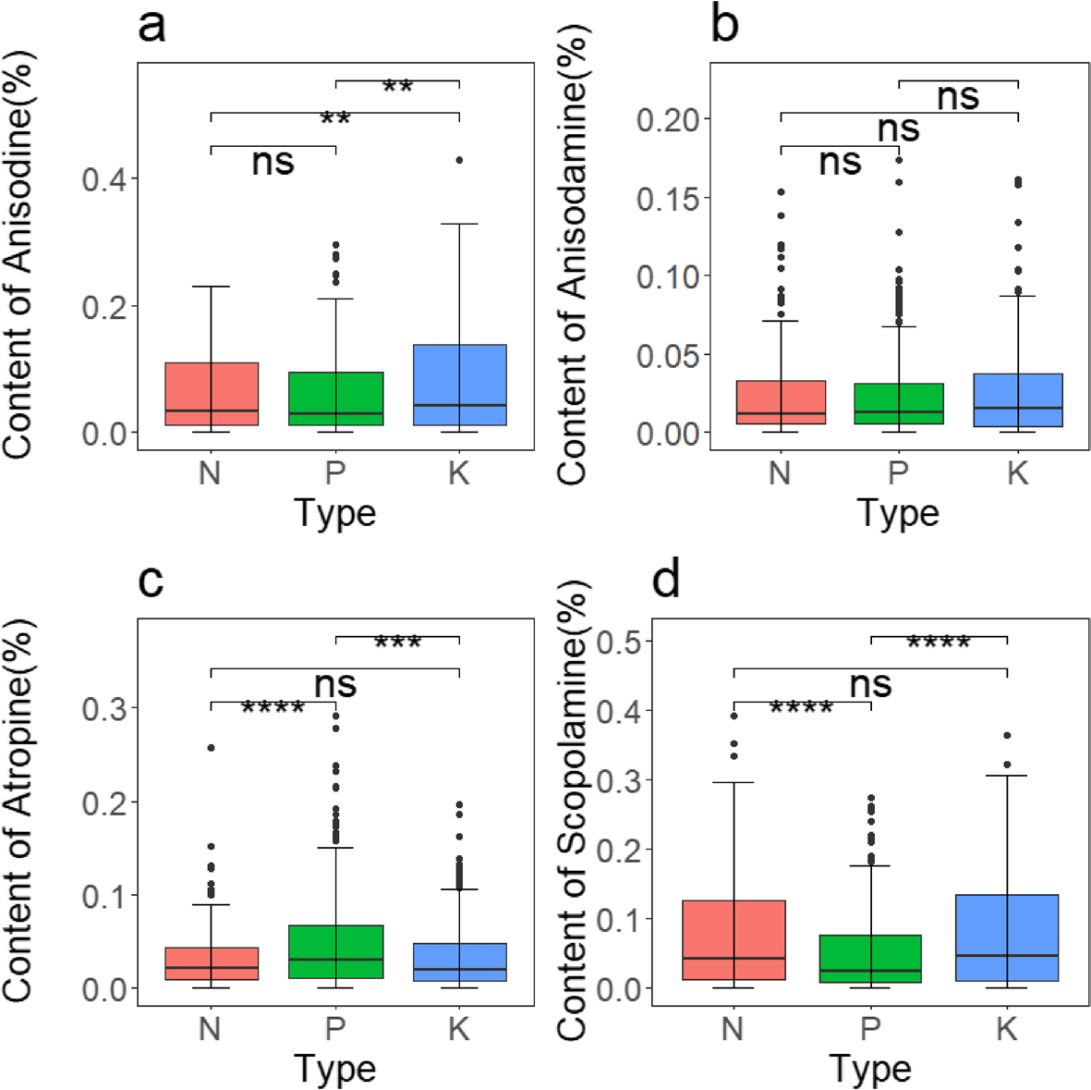
Major alkaloid contents of *A. tanguticus* aboveground parts in response to different nutrients (t-tests were used to determine significant differences at the *P* < 0.05 (*), *P* < 0.01 (**), and *P* < 0.001 (***) levels, with "ns" denoting no significant differences)

To summarize, nitrogen, phosphorus, and potassium additions had varying degrees of influence on the content of major alkaloids in the aboveground parts of *A. tanguticus*. The specific effects were as follows: Anisodine exhibited a pattern of K > P ≈ N; the impact of nitrogen, phosphorus, and potassium additions on aboveground anisodamine content was similar; atropine exhibited a pattern of P > N ≈ K; and scopolamine exhibited a pattern of K ≈ N > P (Fig. 10a, b, c, and d). Moreover, there were statistically significant differences in years, different nutrient additions, growth stages, and their interactions (*P* < 0.001) (Fig. 7, 8, and 9; Table 7).

**Table 7:** Statistical analysis of the main alkaloid contents of aboveground parts of *A. tanguticus* (cm)

	Anisodine		Anisodamine		Atropine		Scopolamine	
	*p-value*	F	*p-value*	F	*p-value*	F	*p-value*	F
Year (2021:2022)	< 0.001	586.75	< 0.001	23.858	< 0.001	208.355	ns	0.602
Period	< 0.001	698.63	< 0.001	478.522	< 0.001	134.075	< 0.001	480.036
Type(N:P:K)	< 0.001	34.848	ns	2.113	< 0.001	31.308	< 0.001	57.879
Treatment	< 0.001	5.747	< 0.001	4.624	< 0.001	9.072	< 0.001	4.947
Year*Treatment	< 0.001	3.521	< 0.05	1.917	< 0.001	5.541	< 0.01	2.066
Treatment*Period	< 0.001	2.659	< 0.001	2.191	< 0.001	3.935	< 0.001	2.344
Year: Type	< 0.01	5.458	ns	1.178	ns	0.261	ns	0.714
Type: Period	< 0.001	8.352	ns	1.558	< 0.001	10.491	< 0.001	9.189

*ns* non-significant

## Seasonal variation in the exogenous regulation of macro-nutrients on the major alkaloids of *A. tanguticus*

**Fig. 11:**
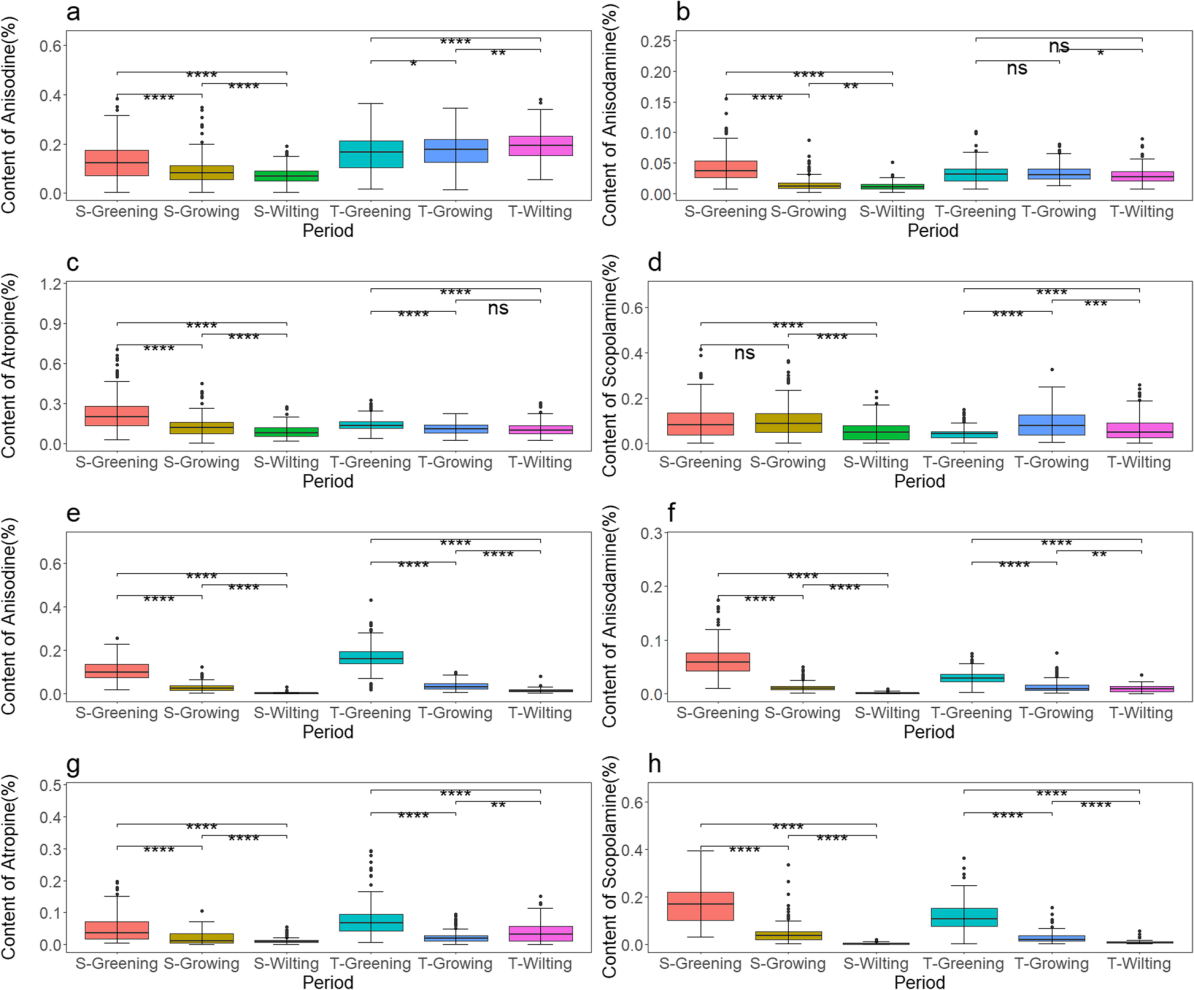
Seasonal effects of different nutrients on the content of major alkaloids of *A. tanguticus* (t-tests were used to determine significant differences at the *P* < 0.05 (*), *P* < 0.01 (**), and *P* < 0.001 (***) levels, with "ns" denoting no significant differences, a, b, c, and d represent major alkaloid content in roots, e, f, g, and h represent major alkaloid content in aboveground parts)

The alkaloid content in the roots of *A. tanguticus* exhibits a certain seasonal variation with its growth. Specifically, the anisodine content significantly decreased during the 2021 growing season and accumulated significantly in 2022 (Fig. 11a). Under nitrogen, phosphorus, and potassium additions, the anisodine content during S-Greening was significantly higher than during S-Wilting. Additionally, the addition of nitrogen and phosphorus resulted in a significant increase in anisodine content from S-Greening to S-Wilting, as shown in Fig. 3a and 4a. However, the addition of potassium did not show any statistically significant effect, as depicted in Fig. 5a. The anisodamine content significantly decreased during the 2021 growing season but remained relatively stable during the 2022 growing season (Fig. 11b). Under the conditions of nitrogen, phosphorus, and potassium additions, the anisodamine content during S-Greening was notably higher than during S-Growing and S-Wilting. Furthermore, the anisodamine content during S-Growing was much higher than during S-Wilting, as shown in Fig. 3b, 4b, and 5b. The atropine content significantly decreased in both years during the growing season, with no significant difference between T-Growing and T-Wilting (Fig. 11c). Under nitrogen and potassium additions, except for no significant difference in atropine content between T-Growing and T-Wilting, there was a significant difference in other growth stages (Fig. 3c, 5c). Under phosphorus addition, atropine content during S-Greening was significantly higher than during S-Growing and S-Wilting, with no significant difference between the other growth stages (Fig. 4c). The scopolamine content displayed a trend of increasing and then decreasing during the two years of growth (Fig. 11d). Under nitrogen, phosphorus, and potassium additions, the scopolamine content during S-Greening and S-Growing was significantly different from S-Wilting, and T-Greening was significantly different from T-Growing, as shown in Fig. 3d, Fig. 4d, and Fig. 5d.

Similarly, the aboveground alkaloid content of *A. tanguticus* also shows some seasonal variation. Particularly, the content of anisodine, anisodamine, atropine, and scopolamine significantly decreases with the growth season, reaching its highest during the greening period (Fig. 11e-h). Under nitrogen, phosphorus, and potassium nutrient additions, the aboveground content of anisodine and scopolamine was significantly different between each growth stage (Fig. 7a, d; Fig. 8a, d; Fig. 9a, d), while anisodamine and atropine, except for no significant difference between T-Growing and T-Wilting, were significantly different between other growth stages, with atropine also not being significant between S-Growing and S-Wilting (Fig. 7b, c; Fig. 8b, c; Fig. 9b, c).

Furthermore, we found that nutrient additions in the greening period of *A. tanguticus* coordinate and regulate the alkaloid content between its aboveground and root parts. During the S-Greening period, the concentration of anisodamine and scopolamine in the aboveground portion was higher than in the roots (Fig. 11b, d, f, and h). Phosphorus addition also increased the content of anisodine aboveground compared to the roots during S-Greening, as seen in Fig. 4a and 8a. Additionally, during T-Greening, phosphorus addition increased the content of anisodine, anisodamine, atropine, and scopolamine aboveground compared to the roots, and the total content of these four alkaloids aboveground was 0.65 g/plant, while underground was 0.39 g/plant. Meanwhile, potassium addition also showed a trend of increasing the content of anisodine, anisodamine, and scopolamine aboveground compared to the roots (Fig. 5a, b, and d; Fig. 9a, b, and d). Nitrogen addition only resulted in a higher scopolamine content aboveground compared to the roots (Fig. 3d, 7d). This not only reveals the dynamic changes in alkaloid content in different parts of *A. tanguticus* but also highlights the regulatory role of nitrogen, phosphorus, and potassium additions.

## Exogenous regulation of macro-nutrients affects the accumulation and yield *of major* alkaloids in *A. tanguticus*

**Fig. 12:**
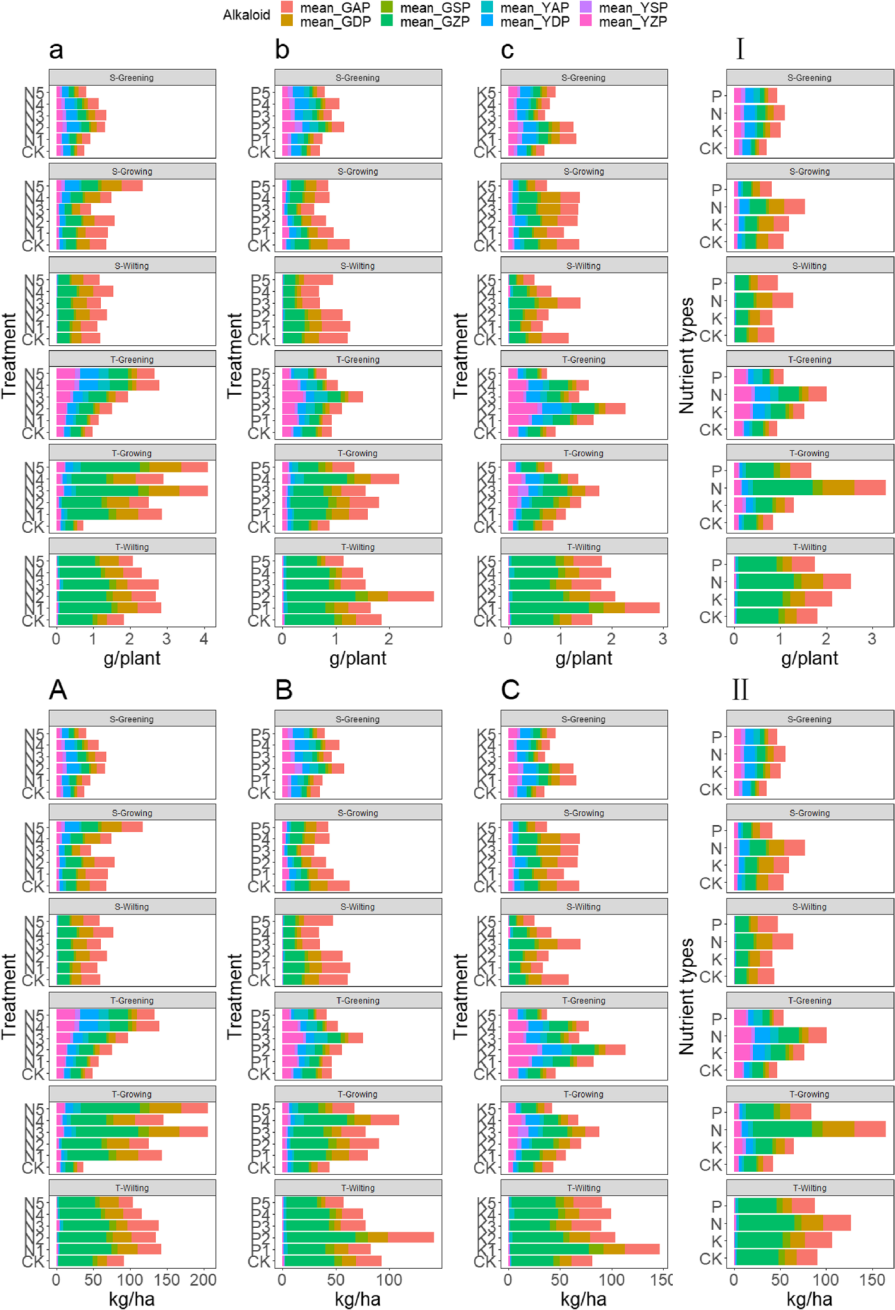
Accumulation and yield of *A. tanguticus* alkaloids (a, b, and c represent the accumulation of the major alkaloid content of *A. tanguticus* per unit area under the addition of nitrogen, phosphorus, and potassium, respectively. I represents the effect of nutrient type on the accumulation of major alkaloid content in *A. tanguticus*. A, B, and C represent the accumulation of major alkaloid content per unit area of *A. tanguticus* under the addition of nitrogen, phosphorus, and potassium, respectively. II represents the effect of nutrient type on the accumulation of major alkaloid content per unit area of *A. tanguticus*)

The analysis of alkaloid accumulation per plant and alkaloid yield per unit area in *A. tanguticus*, as depicted in Fig. 12, demonstrated a progressive increase in alkaloid accumulation throughout the growth season when nitrogen, phosphorus, and potassium were added. The highest accumulation was observed with the addition of nitrogen. During T-Growing, the alkaloid accumulation per plant reached 4.106 g/plant at N3 level, and the unit area alkaloid yield accumulation reached 205.79 kg/ha (Fig. 12a), which was 4.59 times, 0.88 times, and 1.33 times higher than CK, phosphorus addition, and potassium addition, respectively. The root accounted for 84.24% of the total accumulation. The optimal unit area alkaloid yield under phosphorus and potassium addition during S-Wilting at P2 and K1 levels reached 142.18 kg/ha and 146.91 kg/ha, respectively, with the root accounting for 97% to 99% of the accumulation, as shown in Fig. 12B and 12C. Nevertheless, the concentration of alkaloids per unit area in the aboveground portion exhibited a declining pattern during the growth season, particularly in the second year. During the greening period, the alkaloid accumulation per plant in the aboveground part under nitrogen, phosphorus, and potassium additions was 1.44 g/plant, 0.85 g/plant, and 1.22 g/plant, respectively (Fig. 12a, b, and c). The unit area alkaloid yields reached 72.36 kg/ha, 42.48 kg/ha, and 61.21 kg/ha, respectively, decreasing by 55.19%, 53.10%, and 43.84% during the growing season, as shown in Fig. 12A, B, and C.

In addition, the analysis of nutrient type indicated that nitrogen had a greater impact on alkaloid accumulation per plant and per unit area compared to potassium and phosphorus, as shown in Fig. 12I and II. Nitrogen had a promoting effect on the accumulation and yield of alkaloids, with the highest accumulation observed in the root and aboveground part of anisodine under nitrogen addition, reaching 57.82 kg/ha (1.154 g/plant) and 16.23 kg/ha (0.324 g/plant), respectively, which was the highest proportion among the four alkaloids. Following the figures. presented (12a, A), it can be shown that atropine and scopolamine were the next most abundant, while anisodamine had the least proportion. It is noteworthy that during the greening period, the effect of phosphorus addition on atropine and anisodamine in the aboveground part was superior to nitrogen and potassium, with atropine reaching 8.65 kg/ha (0.173 g/plant) in T-Greening, an increase of 6.60% and 38.96% compared to nitrogen and potassium, respectively. In S-Greening, the aboveground part of anisodamine reached 4.53 kg/ha (0.090 g/plant), an increase of 13.38% and 79.51% compared to nitrogen and potassium, respectively (Fig. 12A, B, C, I, II).

## Optimal analysis based on response surface and c-d production function

The rsm function was utilized to establish a response surface mode: ${\text{Alkaloid Yiled = Period}}+ \text{SO} ({\text{N}}{, }{\text{P}}{, }{\text{K}})$. The model comprises the response variable Alkaloid Yiled and the independent variables Period and SO(*N*, *P*, *K*), with SO representing a quadratic polynomial model incorporating linear, quadratic, and interaction terms. The results demonstrate that the coefficients of the independent variables exhibit statistical significance, indicating their impact on the response variable. The model’s coefficient of determination *R*^2^ is 0.6139, with *p* < 0.001, indicating that the model explains 61.39% of the variability in the response variable and is overall significant (Supplementary Table 4). In addition, the "lack of fit" is not significant (*p-value* 0.9558), indicating a good fit to the data. Based on the model results, we obtained the final equation as follows:

$$\begin{array}{c}\text{alkaloid yiled}=33.89+12.09\times S\_\text{Growing}+4.14\times S\_\text{Wilting}+23.56\times T\_\text{Greening}+\\ 46.10\times T\_\text{Growning}+55.41\times T\_\text{Wilting}+0.31\times N+0.03\times P+0.36\times\\ K\text{-0.0005}\times N^2\text{-0.00003}\times P^2\text{-0.002}\times K^2\end{array}$$

Based on the given equation, the coefficient of the independent variable Period’indicates that various growth stages have distinct impacts on alkaloid yield. Harvesting and nutrient input during the T-Growing (46.10) and T-Wilting (55.41) periods should be emphasized, as they have the most significant positive impact on alkaloid yield. The coefficients of the independent variables *N*, *P*, and *K*, and their quadratic terms suggest that adjusting the amounts of different nutrients can optimize alkaloid yield. Specifically, increasing nitrogen and potassium in moderate amounts may have a positive effect on alkaloid yield, while adding phosphorus in a moderate range could also positively impact yield. Additionally, the presence of quadratic terms in the equation indicates that once nutrient concentrations surpass a specific threshold, the production of alkaloids may decline. Therefore, it is crucial to refrain from excessive fertilization and maintain nutrient levels within a moderate range in order to optimize alkaloid production.

Moreover, the equation’s coefficients reveal that the T-Wilting period has the greatest influence on alkaloid yield, as indicated by a coefficient of 55.41, which is significantly greater than coefficients associated with other growth periods. Thus, it can be inferred that the T-Wilting period is the optimal harvesting time for alkaloid yield. According to the model results, the parameter estimate for N is 0.31, which is substantially smaller than the parameter estimate for K (0.36). Although the effect of N is relatively smaller, it is statistically more significant. This is evident from the smaller standard error (N: 0.09, K: 0.10), larger T-*value* (N: 3.61, K: 2.65), and smaller *p-value* (N: 0.000486, K: 0.009435). These results highlight the more significant and reliable influence of N in the model (Supplementary Table 4, Supplementary Fig. 4).

The model suggests that the ideal amounts of nutrient input are as follows: *N* = 324.11 kg/ha, *P *= 0 kg/ha, K = 101.44 kg/ha. According to this information, the projected highest possible amount of alkaloid produced is 157.78 kg per hectare. This forecast can be utilized to ascertain the optimal time for harvesting, optimize the application of nutrients, and implement other management strategies to increase crop productivity. Nevertheless, it is important to take caution when interpreting this prediction, as the actual outcomes may be influenced by unaccounted factors.

We have chosen the most suitable time for harvesting, known as T-Wilting and have normalized all the measured indicators by adding the appropriate amount of nitrogen (N). This is done in preparation for the "three-stage modeling" process. Initially, linear regression was utilized to analyze the correlation between all factors and alkaloid yield, resulting in coefficient estimates (*R*^2^ = 0.98, F = 104; *p* < 0.01). Following that, LASSO regression was performed, incorporating L1 regularization to find crucial factors that have a large impact on alkaloid yield while also reducing the coefficients of less relevant factors to zero. In this case, the coefficients for plant height, root diameter, and aboveground fraction of anisodine were reduced to zero and subsequently removed from the analysis (Supplementary Fig. 5). Linear regression was conducted once more, this time utilizing the variables chosen via LASSO regression (F = 121.5, F = 153.4; *p* < 0.01). The results identified several significant indicators associated with alkaloid yield, including root dry weight, root anisodine, root scopolamine, root atropine, aboveground scopolamine, and aboveground atropine (Supplementary Table 5). We utilized the aforementioned indicators in the context of the Cobb–Douglas production function, a widely employed economic model for assessing the influence of input components on output. Additionally, it is utilized to examine the impact of agricultural practices on crop output, evaluate the effectiveness of agricultural production, investigate the integration of production elements, and develop agricultural policies [62, 63]. Regression was performed with the obtained indicators, and in the parameter estimates of the regression model (*R*^2^ = 0.95, F = 121.5, *p* < 0.01), we assessed the impact of different indicators on the alkaloid yield of *A. tanguticus*. The intercept term coefficient was close to zero, and positive elasticity coefficients indicated that an increase in the respective indicator would lead to an increase in alkaloid yield. The Cobb–Douglas production function obtained is as follows:

$$\begin{array}{cc} \text{alkaloid yiled}_{\mathrm{T}\_\text{wilting}\_\mathrm{N}}=root\,dry\,biomass^{0.88}\bullet root\,Ani.^{0.19}\bullet root\,Sco.^{0.27}\bullet\\ root\,Atr.^{0.23}\bullet above\,SCo.^{0.14}\bullet above\,Atr.^{0.1}\end{array}$$

This function elucidates the correlations among various production input parameters, namely root dry weight, root anisodine, root scopolamine, root atropine, aboveground scopolamine, aboveground atropine, and the resulting alkaloid yield. The production factor coefficients illustrate their influence on the alkaloid yield. For instance, a coefficient of 0.88 signifies the impact of an augmentation in root biomass on the production of alkaloids. A 1% increase in root biomass will result in a 0.88% increase in alkaloid yield, indicating a significant positive correlation between root biomass and alkaloid yield.

## Discussion

### Impact of nitrogen addition on the growth and quality of *A. tanguticus*

In our study, nitrogen addition exhibited a significant increasing effect on the plant height, root length, and root diameter of *A. tanguticus*. The impact was particularly pronounced at high nitrogen levels during the growing period, indicating a responsive adjustment of plant height and root growth to nitrogen absorption. Nitrogen plays a key role in plants and regulates plant growth and development mainly by affecting protein and nucleic acid synthesis [64, 65]. Different nitrogen levels significantly affect the allocation ratio of nitrogen in non-photosynthetic and photosynthetic systems, thereby influencing plant growth and yield [66, 67]. Moreover, various nitrogen levels promoted the accumulation of biomass in different growth stages of *A. tanguticus*. For instance, at the N5 level (375 kg/ha) during T-Greening, the aboveground biomass increased by 1.4 times, and the root biomass during T-Growing at the N5 level increased by 3.78 times, reaching the maximum root dry matter ratio. These suggest that a sufficient nitrogen supply promotes early nutritional growth and accelerates the accumulation of root biomass during the vigorous growth period. This finding aligns with previous studies on nitrogen-sensitive medicinal plants [68–70]. Simultaneously, the response of the root-to-shoot ratio in *A. tanguticus* to nitrogen levels showed a trend of being higher at low concentrations than at high concentrations. This may align with the nitrogen response strategy observed in some plants, such as *Panax notoginseng* and *Globe artichoke*, where low nitrogen prompts a foraging strategy in roots, while high nitrogen invokes a survival strategy by inhibiting root growth [71–73]. Under high nitrogen conditions, plants sacrifice root biomass to allocate more biomass aboveground, leading to a promotion of aboveground biomass accumulation with less impact on belowground biomass [74–76]. However, it is crucial to note that excessive nitrogen may result in a relative dilution of active plant compounds, adversely affecting quality [69, 77]. Our study found that varying nitrogen levels enhanced the accumulation of major alkaloids in both the root and aboveground parts of *A. tanguticus*, with the accumulation effect being lower at high nitrogen levels than at moderate levels. This suggests that excessive nitrogen addition weakens the positive regulatory effect of nitrogen on alkaloid accumulation in *A. tanguticus*. While high nitrogen promotes biomass accumulation, it diminishes alkaloid accumulation. This phenomenon has been observed in other medicinal plants, including *Tripterygium wilfordi*i, *Panax notoginseng*, ginseng and *Panax quinquefolius* [36, 71, 78, 79]. In addition, during the greening period, nitrogen levels (225–300 kg/ha) were more favorable for the accumulation of root alkaloids, while the N4 level (300 kg/ha) was more conducive to the increase in aboveground alkaloid content. This might be attributed to roots being the primary site for the biosynthesis of tropane alkaloids, and with plant growth, medium to high nitrogen levels (300 kg/ha) promote the transfer of alkaloids from roots to tender aboveground tissue [19, 80]. This further emphasizes the crucial regulatory role of nitrogen in the metabolism of tropane alkaloids in *A. tanguticus*, revealing the synergistic action of roots and aboveground parts in alkaloid synthesis and transformation. It is noteworthy that the highest accumulation of alkaloid yield per unit area in *A. tanguticus* was achieved at the N level of T-Growing (225 kg/ha), reaching 205.79 kg/ha, an increase of 3.6 times compared to the control (CK). Importantly, at this level, root accumulation accounted for 84.24%, and aboveground accumulation accounted for 15.76%. This indicates that nitrogen addition not only promotes the accumulation of alkaloids in the roots of *A. tanguticus* but also enhances the accumulation of alkaloids in its aboveground parts. Therefore, during the harvesting of *A. tanguticus*, consideration should also be given to the medicinal value of its aboveground parts to ensure the optimal utilization of resources.

## Impact of phosphorus addition on the growth and quality of *A. tanguticus*

Adequate phosphorus supply contributes to maintaining normal plant growth and promoting the development of both roots and aboveground parts [81, 82]. In this study, moderate phosphorus addition (900 kg/ha) significantly increased the plant height, root length, and root diameter of *A. tanguticus*. This enhancement is attributed to phosphorus being a crucial component in ATP synthesis, with ATP being the primary driving force for energy transfer in plants [83]. The moderate phosphorus level likely provided sufficient phosphorus, prompting more efficient photosynthesis and energy production, leading to a significant improvement in growth traits [39]. On the other hand, low phosphorus addition (600 kg/ha or 750 kg/ha) significantly increased the root biomass of *A. tanguticus*. Meanwhile, the root-to-shoot ratio was higher at low concentrations (600 kg/ha or 750 kg/ha) than at high concentrations (1200 kg/ha). This reflects the plant’s physiological adaptation to phosphorus demands. Under low phosphorus conditions, increasing root biomass enhances the absorption of limited phosphorus resources, representing an adaptive strategy in plants [47, 84, 85]. However, the impact of different phosphorus levels on the aboveground biomass of *A. tanguticus* was relatively weak. This may be due to the plant’s relatively low demand for phosphorus in aboveground parts or the influence of other environmental factors, resulting in less pronounced effects of phosphorus on aboveground biomass. In summary, the results of this study reveal the regulatory role of phosphorus in the growth and biomass allocation of *A. tanguticus*. Phosphorus has also been found to play a crucial role in the healthy development of roots and biomass accumulation in medicinal plants such as *Lupinus angustifolius*, *Dendrobium officinale*, and *Bupleurum chinense* [45, 49, 52]. Previous studies have demonstrated that appropriate phosphorus addition helps improve the quality of medicinal plants while ensuring economic yield [86, 87]. However, in our study, different levels of phosphorus addition did not significantly affect the content of major alkaloids, including anisodine, atropine, and scopolamine, in the roots of *A. tanguticus*. It is noteworthy that, with increasing phosphorus content, there was a decreasing trend in the content of these alkaloids. In contrast, low phosphorus significantly increased the content of major alkaloids in the aboveground parts of *A. tanguticus*, with atropine showing a particularly prominent increase, approximately six times higher than the control (CK). These results indicate that low-concentration phosphorus addition is more effective in promoting the accumulation of alkaloids in the aboveground parts of *A. tanguticus*. This phenomenon has also been observed in *Bupleurum chinense*, *Dendrobium officinale*, and *Artemisia annua* [52, 88], where low phosphorus enhances the production of certain secondary metabolites in plants. This is attributed to the complex regulation of phosphorus entry into the plant body through gene expression networks, with low phosphorus stress upregulating the genes of key enzymes in the biosynthesis of secondary metabolite [88, 89]. Additionally, genes responding to low phosphorus stress and phosphoric acid transport proteins in plants increase with phosphorus deficiency [90, 91]. Therefore, phosphorus management in the cultivation of *A. tanguticus* requires careful application. Adding a certain amount of phosphorus within a moderate range can enhance both yield and quality, avoiding the negative impacts associated with excessive phosphorus.

## Impact of potassium addition on the growth and quality of *A. tanguticus*

Adequate potassium supply enhances a plant’s water regulation capacity, photosynthetic efficiency, and resistance to environmental stress, thereby increasing the biomass of medicinal plants and boosting the accumulation of medicinal compounds [92, 93]. In this study, low concentrations of potassium (75 kg/ha or 112.5 kg/ha) promoted the growth of *A. tanguticus*, particularly in plant height during the growing season, root length in the greening period, and root diameter during the wilting period, showing significant effects. Similarly, low potassium levels increased both root and aboveground biomass, indicating that low-concentration potassium addition provided the necessary nutrients for the growth of *A. tanguticus*, promoting development during critical growth stages. Previous research has shown that adequate potassium also promotes the growth and biomass accumulation of medicinal plants such as *Rheum tanguticum*, *Salvia miltiorrhiza*, and *Fritillaria thunbergii* [56, 93, 94]. However, high-concentration potassium addition inhibited the growth and biomass accumulation of *A. tanguticus*. This inhibition may be due to the competition and imbalance among mineral elements, such as calcium ions and magnesium ions, caused by high concentrations of potassium [95–97]. Additionally, high concentrations of potassium may lead to an osmotic regulation imbalance, increasing the difference in osmotic pressure between intracellular and extracellular environments and affecting water absorption and transport [98–100]. Therefore, moderate potassium addition has a promoting effect on the growth and biomass accumulation of the medicinal plant *A. tanguticus*. Furthermore, in our study, the addition of 75 kg/ha potassium significantly increased the content of anisodine, atropine, and scopolamine in the roots of *A. tanguticus* during the regreening period. Simultaneously, it increased the content of atropine in the aboveground parts. Conversely, high potassium addition had an inhibitory effect on the accumulation of alkaloids in *A. tanguticus*. This could be attributed to the crucial role of potassium in the synthesis of tropane alkaloids. Previous studies have found that Fe^2+^ significantly activates the key enzyme involved in tropane alkaloid synthesis—hyoscyamine 6β-hydroxylase—while divalent cations such as Mn^2+^ and Co^2+^ strongly inhibit this enzyme [101, 102]. Moreover, lower concentrations of Ca^2+^ can decrease the synthesis of scopolamine [103]. Potassium, existing in the ionic state (K^+^), plays a role in maintaining ion balance within cells, and it may indirectly influence the synthesis of key enzymes in the tropane alkaloid synthesis process through competition, absorption, transportation, and antagonistic effects among these ions [26, 54, 55]. Recent research also indicates that potassium addition increases intermediate products of the tricarboxylic acid (TCA) cycle and enhances mobile nitrogen reservoirs, such as putrescine and asparagine (Asn), an amino acid [104, 105]. Interestingly, putrescine serves as a precursor for tropane alkaloid synthesis [22]. Therefore, maintaining an appropriate level of potassium is crucial for the physiological functions and quality of medicinal components in *A. tanguticus*. It is noteworthy that, in this study, potassium addition led to the maximum unit area alkaloid yield of 146.91 kg/ha during the wilting period at a low potassium level (75 kg/ha), where root accumulation accounted for 97% to 99%.

To summarize, the addition of nitrogen, phosphorus, and potassium regulates the growth, development, and medicinal quality formation of the medicinal plant *A. tanguticus* to varying degrees, with the order of effectiveness being nitrogen > potassium > phosphorus. Response surface analysis incorporating all nutrient additions and their interactions with alkaloid yield across different growth stages of *A. tanguticus* yielded consistent results. Practical implications suggest that increasing nitrogen and potassium within appropriate limits may have a positive impact on alkaloid yield, while moderate phosphorus addition could positively influence yield. This is further confirmed by Fig. 6a, b, c, and d, Fig. 10a, b, c, and d. Moreover, based on our response surface results, the wilting period in the third year after transplantation is identified as the optimal harvest period for this experiment. Alkaloid accumulation correlates with the accumulation of *A. tanguticus* root biomass, while aboveground parts are shed as the growing season progresses (Fig. 11). Subsequently, in the analysis of the Douglas production function, the optimal harvest period T-Wilting, with optimal nutrient N, indicates that, for cultivating *A. tanguticus* in this region, attention should be focused on the third wilting period and the accumulation of root biomass, followed by the accumulation of scopolamine and atropine content in the roots.

## Conclusions

Nutrient addition significantly influences the growth and alkaloid content of *A. tanguticus*. Specifically, high nitrogen (375 kg/ha) and low potassium (75, 112.5 kg/ha) significantly promote multiple growth indicators, with high nitrogen particularly demonstrating a pronounced stimulatory effect on the overall biomass of *A. tanguticus*. In contrast, the impact of phosphorus is relatively weaker, especially regarding its insignificant effect on aboveground biomass. Concerning alkaloid content, moderate nitrogen (225 kg/ha) shows a significant enhancement, followed by low potassium (75 kg/ha), while phosphorus has a minor impact on the increase in alkaloid content. It is noteworthy that phosphorus addition, at specific stages, exhibits a decreasing trend in the content of scopolamine in the roots, concurrently with an increase in phosphorus concentration leading to a decline in aboveground scopolamine content. Furthermore, during the regreening period of *A. tanguticus*, nitrogen (300 kg/ha), phosphorus (900 kg/ha), and potassium (75 kg/ha) additions significantly increase the content of aboveground atropine, with the order of the enhancing effect being P > K > N, and it exhibits the highest increment compared to other alkaloids. The maximum alkaloid accumulation per unit area for *A. tanguticus* follows the order of N > K > P, with nitrogen addition at the middle nitrogen level (225 kg/ha) during the T-Growing period reaching 205.79 kg/ha, potassium addition at the low potassium level (75 kg/ha) during the S-Wilting period reaching 146.91 kg/ha, and phosphorus addition at the low phosphorus level (750 kg/ha) during the S-Wilting period reaching 142.18 kg/ha. Response surface model analysis indicates that the T-Wilting period is the optimal harvesting time, with the optimal nutrient combination being N = 324.11 kg/ha, P = 0 kg/ha, K = 101.44 kg/ha, and the predicted maximum economically viable total alkaloid yield being 157.78 kg/ha. Results from the Douglas production function reveal that, when maximizing the total alkaloid production in the region, focusing on root biomass, scopolamine, and atropine content in the roots is crucial. In conclusion, by judiciously selecting and regulating the amounts of different nutrient additions, optimal accumulation of alkaloid yield per unit area can be achieved, providing a scientific basis for the efficient cultivation of *A. tanguticus*.

### Supplementary Information

Supplementary Material 1.

## Data Availability

Data will be made available on request.
