# Supplementary material for: Exogenous regulation of macronutrients promotes the accumulation of alkaloid yield in anisodus tanguticus (Maxim.) pascher
Source: BMC Plant Biol. 2024 Jun 26;24:602. doi: 10.1186/s12870-024-05299-8 (PMC11201296; doi:10.1186/s12870-024-05299-8)
Supplement: Supplementary file 1 — Supplementary Material 1. [file 12870_2024_5299_MOESM1_ESM.docx]

**Exogenous Regulation of Macronutrients Promotes the Accumulation of Alkaloid Yield in *Anisodus tanguticus* (Maxim.) Pascher**

Na Liu ^1, 2^, Chen Chen ^3^, Bo Wang ^1^, Xiaoyun Wang ^1, 2^, Dengshan Zhang ^2 *^, Guoying Zhou ^1 *^

1 CAS Key Laboratory of Tibetan Medicine Research, Northwest Institute of Plateau Biology, Xining 810008, China

2 State Key Laboratory of Plateau Ecology and Agriculture, Qinghai University, Xining 810016, China

3 College of Life Sciences, Huaibei Normal University, Huaibei, 235000, China

*Corresponding author: Dr. Guoying Zhou & Dr. Dengshan Zhang

E-mail addresses: [zhougy@nwipb.cas.cn](mailto:zhougy@nwipb.cas.cn) (G.Y. Zhou), [dshzhang@bnu.edu.cn](mailto:dshzhang@bnu.edu.cn) (D.S. Zhang)

Tel: +86-971-6159630

Fax: +86-971-6143282

Address: 23# Xinning Road, Xining, Qinghai, P. R. China 810008

Table S1 Moisture of *A. tanguticus* samples (%)

| Period | Root | Left |  |
| --- | --- | --- | --- |
| S-Green | 0.078 | 0.0633 |  |
| S-Growth | 0.0872 | 0.0834 |  |
| S-Wilting | 0.0662 | 0.0583 |  |
| T-Green | 0.0766 | 0.0645 |  |
| T-Growth | 0.0864 | 0.0825 |  |
| T-Wilting | 0.0658 | 0.0595 |  |
| Values are means of three replicates | | | |

Table S2 Alkaloid Extraction and Determination Related Instruments

| Instruments | Source |
| --- | --- |
| Agilent-1260 System High Performance Liquid Chromatography | Agilent Technologies Inc. (USA) |
| Agilent 5HC-C18 Columns | Agilent Technologies Inc. (USA) |
| Milli-Q Ultrapure Water Meter | Merck KGaA, Darmstadt (Germany) |
| KH-500DE CNC Ultrasonic Cleaner | Kunshan Wo Chuang Ultrasonic Instrument Co. |
| BCD-639WKPZM Electric Blast Drying Oven | Tianjin Tester Instrument Co., Ltd (China) |
| ME204 Electronic Precision Balance | METTLER TOLEDO Instruments (Shanghai) Co. |
| R1001VN Rotary Evaporator | Zhengzhou Great Wall Science, Industry and Trade Co. |
| HH-6 Type Electrothermal Thermostatic Water Bath | Beijing Kewei Yongxing Co., Ltd. (China) |
| FW135 Pulverizer | Shanghai Jingqi Instrument Co. |

Table S3 Reagents and Medicines Related to Alkaloid Extraction and Determination

| Reagents and Medicines | Source |
| --- | --- |
| Methanol (chromatographically pure) | Shandong Yuwang Industrial Co., Ltd. Chemical Branch (China) |
| Acetonitrile (chromatographically pure) | Shandong Yuwang Industrial Co., Ltd. Chemical Branch (China) |
| Phosphoric acid (analytically pure) | Sinopharm Chemical Reagent Co. |
| Trichloromethane (analytically pure) | Baiyin Liangyou Chemical Reagent Co. |
| Ammonia (analytically pure) | Tianjin Damao Chemical Reagent Factory (China) |
| Potassium dihydrogen phosphate (analytically pure) | Sinopharm Chemical Reagent Co. |
| Triethylamine (analytically pure) | Tianjin Fuyu Fine Chemical Co. |
| Anisodamine hydrobromide standard (55449-49-5) | Beijing Yihua Tongbiao Technology Co. |
| Scopolamine hydrobromide standard（114-49-8） | Beijing Yihua Tongbiao Technology Co. |
| Anisodine hydrobromide standard（78804-17-8） | Beijing Yihua Tongbiao Technology Co. |
| Atropine sulfate standard（5908-99-6） | Beijing Yihua Tongbiao Technology Co. |

Table S4 Results of response surface modeling

|  |  | Estimate | Std. Error | t-value | *P*-value | |
| --- | --- | --- | --- | --- | --- | --- |
| Model coefficients | (Intercept) | 33.89 | 7.24 | 4.68 | 0.000 | ******* |
|  | S-Growing | 12.09 | 7.76 | 1.56 | 0.122 |  |
|  | S-Wilting | 4.14 | 7.76 | 0.53 | 0.594 |  |
|  | T-Greening | 23.56 | 7.76 | 3.04 | 0.003 | ****** |
|  | T-Growing | 46.10 | 7.76 | 5.94 | 0.000 | ******* |
|  | T-Wilting | 55.41 | 7.76 | 7.15 | 0.000 | ******* |
|  | N | 0.31 | 0.09 | 3.61 | 0.000 | ******* |
|  | P | 0.03 | 0.03 | 1.29 | 0.199 |  |
|  | K | 0.36 | 0.10 | 2.65 | 0.009 | ****** |
|  | N^2 | 0.00 | 0.00 | -1.98 | 0.051 |  |
|  | P^2 | 0.00 | 0.00 | -1.34 | 0.182 |  |
|  | K^2 | 0.00 | 0.00 | -2.73 | 0.007 | ****** |
| Model summaries | Multiple R-squared | 0.6123 |  |  |  |  |
|  | Adjusted R-squared | 0.5679 |  |  |  |  |
|  | F-statistic | 13.78 |  |  |  |  |
|  | p-value | ＜0.001 |  |  |  |  |
| Significant: 0 ‘***’ 0.001 ‘**’ 0.01 ‘*’ 0.05 | | | | | | |

Table S5 Results of "Three-stage modeling" after standardization of all measurements in the T-Wilting period under nitrogen addition of *A. tanguticus*

|  |  | Estimate | Std. Error | t-value | *P*-value | |
| --- | --- | --- | --- | --- | --- | --- |
| "One-stage modeling"Model coefficients | (Intercept) | 0.000 | 0.025 | 0.000 | 0.000 |  |
|  | Plant Height | 0.000 | 0.034 | -0.010 | 0.992 |  |
|  | Root Length | 0.017 | 0.032 | 0.524 | 0.604 |  |
|  | Root Diamater | 0.009 | 0.045 | 0.204 | 0.840 |  |
|  | Root Fresh Weight | 0.020 | 0.089 | 0.223 | 0.825 |  |
|  | Left Fresh Weight | 0.112 | 0.099 | 1.133 | 0.267 |  |
|  | Root Dry Weight | 0.800 | 0.084 | 9.516 | 0.000 | *** |
|  | Left Dry Weight | 0.062 | 0.091 | 0.682 | 0.501 |  |
|  | Root Anisodine | 0.203 | 0.031 | 6.477 | 0.000 | *** |
|  | Root Anisodamine | -0.050 | 0.050 | -0.995 | 0.328 |  |
|  | Root Scopolamine | 0.275 | 0.038 | 7.202 | 0.000 | *** |
|  | Root Atropine | 0.289 | 0.048 | 6.059 | 0.000 | *** |
|  | Aboveground Anisodine | -0.063 | 0.056 | -1.141 | 0.263 |  |
|  | Aboveground Anisodamine | -0.029 | 0.066 | -0.440 | 0.663 |  |
|  | Aboveground Scopolamine | 0.192 | 0.093 | 2.069 | 0.048 | * |
|  | Aboveground Atropine | 0.120 | 0.053 | 2.274 | 0.031 | * |
| Model summaries | Multiple R-squared | 0.9818 |  |  |  |  |
|  | Adjusted R-squared | 0.9723 |  |  |  |  |
|  | F-statistic | 104 |  |  |  |  |
|  | p-value | ＜0.001 |  |  |  |  |
| "Two-stage modeling"Model coefficients | (Intercept) | 0.000 | 0.024 | 0.000 | 1.000 |  |
|  | Root Length | 0.021 | 0.031 | 0.675 | 0.504 |  |
|  | Root Fresh Weight | 0.050 | 0.072 | 0.693 | 0.493 |  |
|  | Left Fresh Weight | 0.155 | 0.099 | 1.880 | 0.069 |  |
|  | Root Dry Weight | 0.767 | 0.068 | 11.237 | 0.000 | *** |
|  | Left Dry Weight | 0.021 | 0.078 | 0.273 | 0.786 |  |
|  | Root Anisodine | 0.191 | 0.025 | 7.496 | 0.000 | *** |
|  | Root Anisodamine | -0.070 | 0.044 | -1.606 | 0.118 |  |
|  | Root Scopolamine | 0.262 | 0.032 | 8.187 | 0.000 | *** |
|  | Root Atropine | 0.316 | 0.038 | 8.294 | 0.000 | *** |
|  | Aboveground Scopolamine | 0.110 | 0.029 | 3.789 | 0.001 | *** |
|  | Aboveground Atropine | 0.163 | 0.030 | 5.389 | 0.000 | *** |
| Model summaries | Multiple R-squared | 0.9808 |  |  |  |  |
|  | Adjusted R-squared | 0.9744 |  |  |  |  |
|  | F-statistic | 153.4 |  |  |  |  |
|  | p-value | ＜0.001 |  |  |  |  |
| "Three-stage modeling"Model coefficients | (Intercept) | 0.000 | 0.036 | 0.000 | 1.000 |  |
|  | Root Dry Weight | 0.882 | 0.037 | 23.593 | 0.000 | *** |
|  | Root Anisodine | 0.189 | 0.037 | 5.135 | 0.000 | *** |
|  | Root Scopolamine | 0.274 | 0.039 | 7.041 | 0.000 | *** |
|  | Root Atropine | 0.233 | 0.038 | 6.132 | 0.000 | *** |
|  | Aboveground Scopolamine | 0.139 | 0.041 | 3.374 | 0.002 | * |
|  | Aboveground Atropine | 0.100 | 0.040 | 2.482 | 0.018 | * |
| Model summaries | Multiple R-squared | 0.9505 |  |  |  |  |
|  | Adjusted R-squared | 0.9427 |  |  |  |  |
|  | F-statistic | 121.5 |  |  |  |  |
|  | p-value | ＜0.001 |  |  |  |  |
| Significant: 0 ‘***’ 0.001 ‘**’ 0.01 ‘*’ 0.05 | |  |  |  |  |  |

Fig. S1 Overlapping peaks of some samples' signals


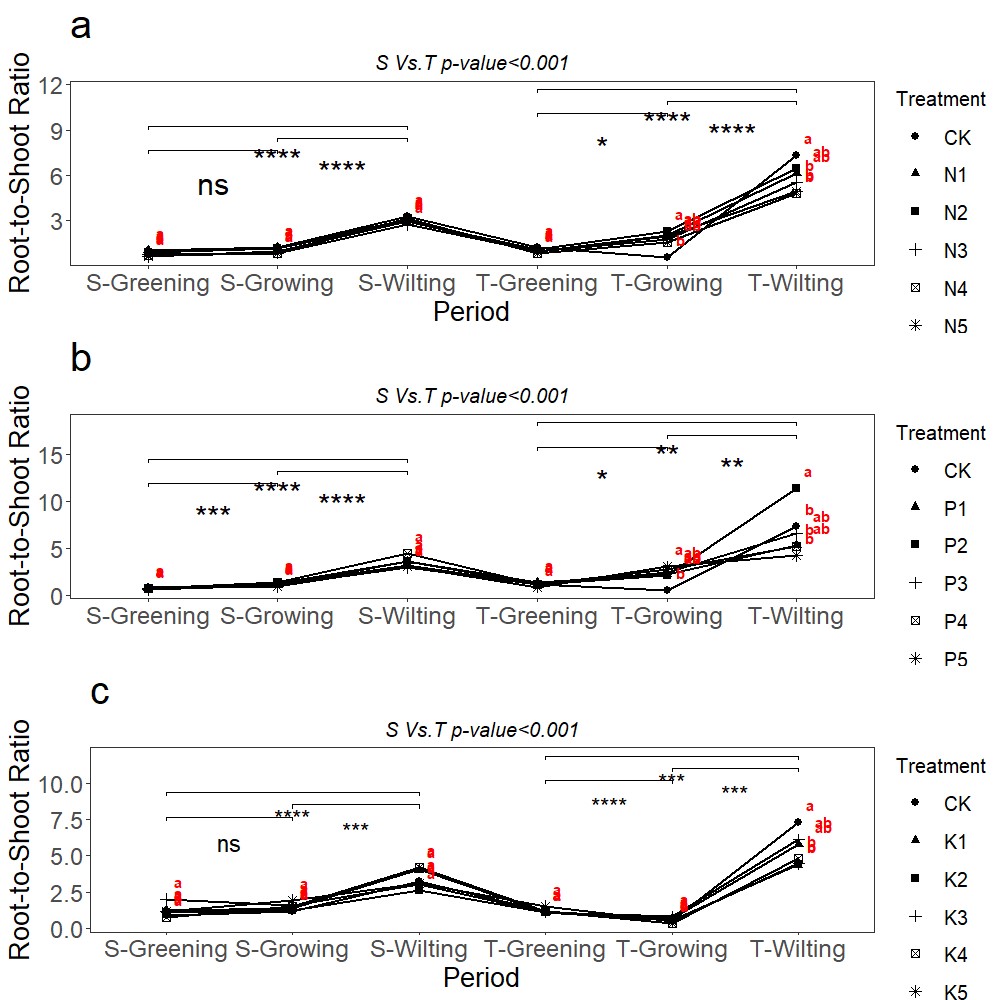


Fig. S2 Effect of nutrient addition on root-crown ratio of *A. tanguticus*


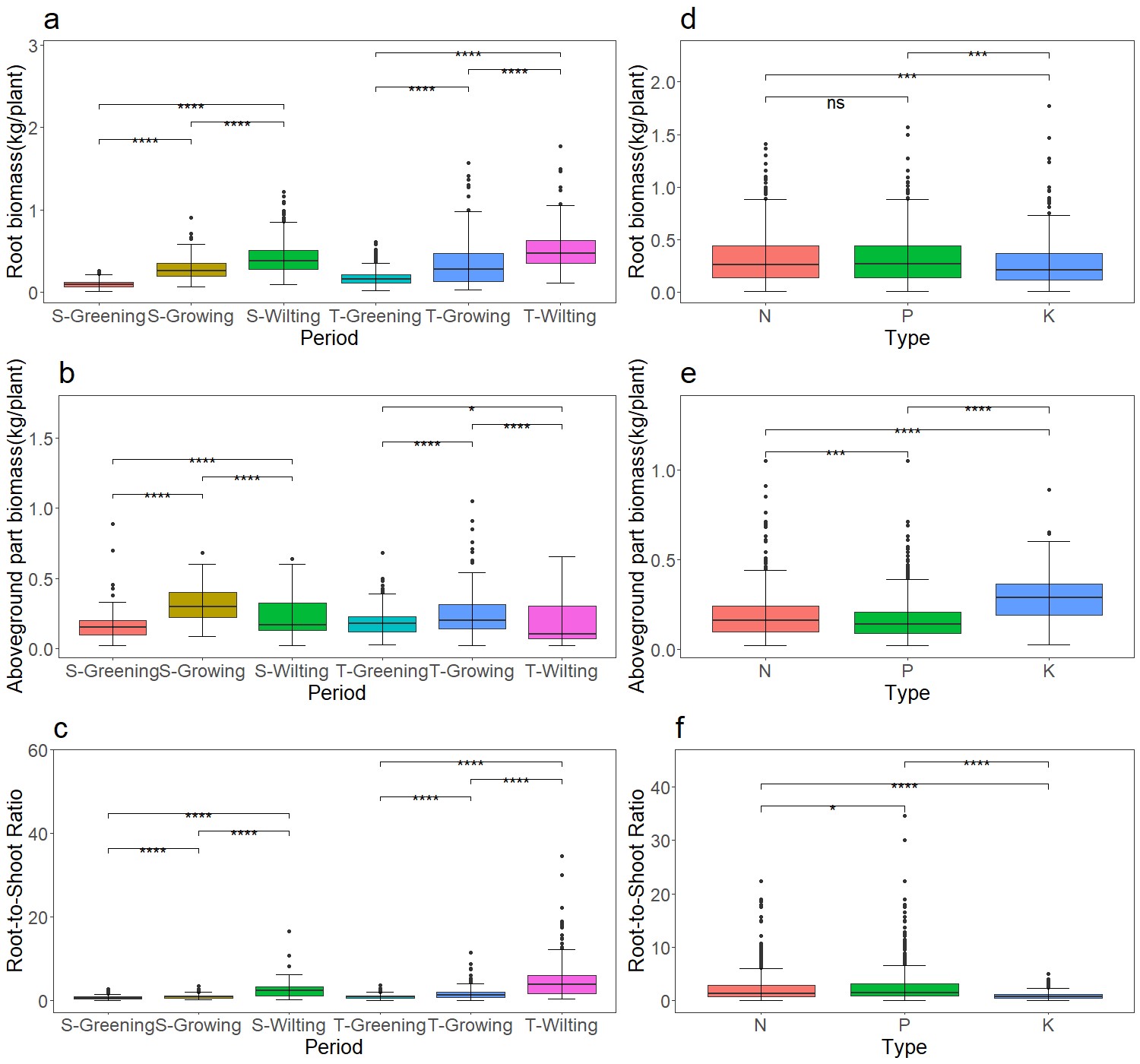


Fig. S3 Effect of nutrient additions on the biomass of roots and above-ground parts of *A. tanguticus*


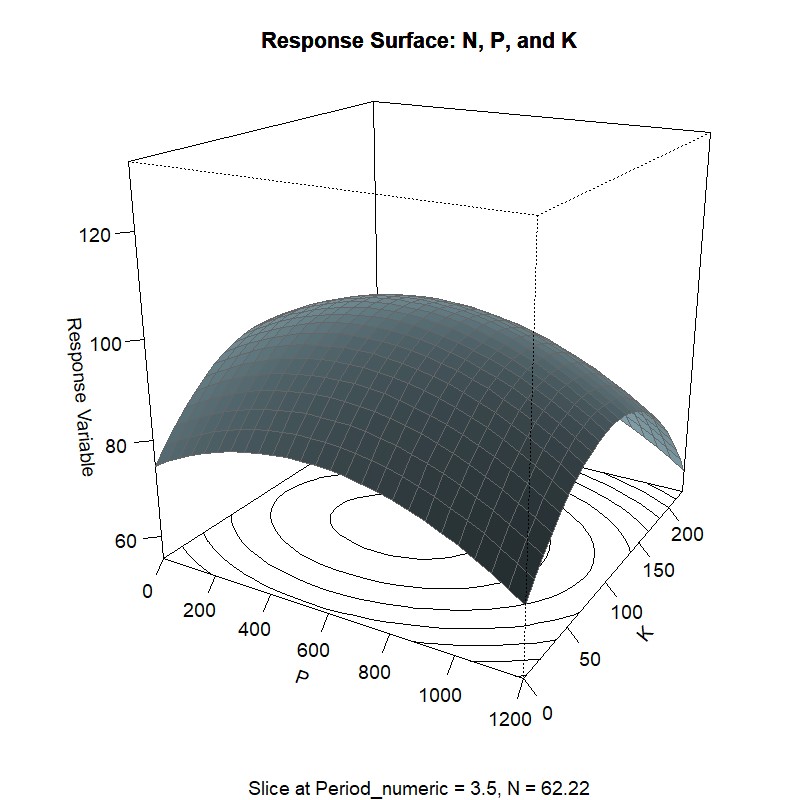


Fig. S4 Response surface result plots for nitrogen, phosphorus and potassium

**
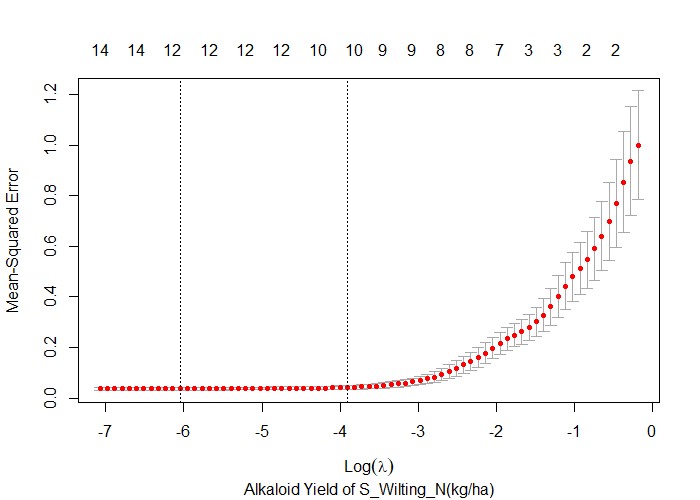
**

Fig. S5 Lasso regression results
